# Adjuvant Chemotherapy Regimens in Resected Biliary Tract Cancers: National, Comparative, Observational Study (TOG/GI‐SAFRADJU‐2501)

**DOI:** 10.1002/ijc.70623

**Published:** 2026-06-29

**Authors:** Erman Akkus, Mehmet Kayaalp, Müzeyyen Aslı Ergözoğlu, Ilgın Koç Kuş, Doğan Bayram, Bilgeşah Kılıçtaş, Emel Ayvaz Güneyin, Ali Kalem, Haydar Temizyürek, Mehmet Mutlu Çatlı, Mehmet Sinan Akarca, Maral Martin Mıldanoğlu, Görkem Turhan, Mahmut Kara, Hacı Arak, Feyza Arslan Tan, Burak Paçacı, Berkay Yeşilyurt, Orhun Akdoğan, Emine Bihter Çetin, Selahattin Çelik, Fatma Keskin Uzundere, Elif Şahin, Zehra Sadak Öcal, Lamia Şeker Can, Bahadır Köylü, Mehmet Cem Fidan, Esmanur Kaplan Tüzün, Teoman Şakalar, Elif Sertesen Çamöz, Haydar Çağatay Yüksel, Onur Yazdan Balçık, Pınar Peker, Mehmet Yılmaz, Şeymanur Ala Enli, Erdem Kölemen, Sibel Oyucu Orhan, Yakup Düzköprü, Mustafa Ersoy, Tuğba Akın Telli, Mesut Yılmaz, Yusuf İlhan, Atila Yıldırım, Ramazan Coşar, Müslih Ürün, Arif Hakan Önder, Murat Araz, Ertuğrul Bayram, Havva Yeşil Çınkır, Ali Alkan, Mustafa Gürbüz, Nilüfer Avcı, Fatma Paksoy Türköz, İsmail Oğuz Kara, Fatih Köse, İlkay Tuğba Ünek, Ahmet Bilici, Öznur Bal, Şuayib Yalçın, Lorenza Rimassa, Hatime Arzu Yaşar

**Affiliations:** ^1^ Department of Medical Oncology Ankara University Faculty of Medicine Ankara Türkiye; ^2^ Cancer Research Institute Ankara University Ankara Türkiye; ^3^ Department of Medical Oncology Cukurova University Faculty of Medicine Adana Türkiye; ^4^ Department of Medical Oncology Hacettepe University Faculty of Medicine Ankara Türkiye; ^5^ Department of Medical Oncology Ankara Bilkent City Hospital Ankara Türkiye; ^6^ Department of Medical Oncology Necmettin Erbakan University Faculty of Medicine Konya Türkiye; ^7^ Department of Medical Oncology Atatürk University Faculty of Medicine Erzurum Türkiye; ^8^ Department of Medical Oncology Gaziantep University Faculty of Medicine Gaziantep Türkiye; ^9^ Department of Medical Oncology Afyonkarahisar University of Health Sciences Afyon Türkiye; ^10^ Department of Medical Oncology Antalya Training and Research Hospital Antalya Türkiye; ^11^ Department of Medical Oncology Dokuz Eylül University Faculty of Medicine İzmir Türkiye; ^12^ Department of Medical Oncology Istanbul Medipol University, Faculty of Medicine İstanbul Türkiye; ^13^ Department of Medical Oncology Karadeniz Technical University Faculty of Medicine Trabzon Türkiye; ^14^ Department of Medical Oncology, Faculty of Medicine Van Yüzüncü Yıl University Van Türkiye; ^15^ Department of Medical Oncology Gaziantep City Hospital Gaziantep Türkiye; ^16^ Department of Medical Oncology İzmir City Hospital İzmir Türkiye; ^17^ Department of Medical Oncology Marmara University Pendik Training and Research Hospital İstanbul Türkiye; ^18^ Department of Medical Oncology Gazi Üniversity Faculty of Medicine Ankara Türkiye; ^19^ Department of Medical Oncology Manisa Celal Bayar University, Faculty of Medicine Manisa Türkiye; ^20^ Department of Medical Oncology Etlik City Hospital Ankara Türkiye; ^21^ Department of Medical Oncology Dicle University Faculty of Medicine Diyarbakır Türkiye; ^22^ Department of Medical Oncology Kocaeli City Hospital Kocaeli Türkiye; ^23^ Department of Medical Oncology İnönü University Turgut Özal Medical Center Malatya Türkiye; ^24^ Department of Medical Oncology, Faculty of Medicine Bezmialem Vakıf University İstanbul Türkiye; ^25^ Department of Medical Oncology Koc University Faculty of Medicine İstanbul Türkiye; ^26^ Department of Medical Oncology İstanbul University Cerrahpasa Medical Faculty İstanbul Türkiye; ^27^ Department of Medical Oncology Gülhane Training and Research Hospital Ankara Türkiye; ^28^ Department of Medical Oncology Kahramanmaraş Necip Fazıl City Hospital Kahramanmaraş Türkiye; ^29^ Department of Medical Oncology Ankara Oncology Hospital Ankara Türkiye; ^30^ Department of Medical Oncology Ege University Faculty of Medicine İzmir Türkiye; ^31^ Department of Medical Oncology, Faculty of Medicine Alanya Alaaddin Keykubat University Antalya Türkiye; ^32^ Department of Medical Oncology Adana City Hospital Adana Türkiye; ^33^ Department of Medical Oncology Eskişehir Osmangazi University Faculty of Medicine Eskişehir Türkiye; ^34^ Department of Medical Oncology Pamukkale University Faculty of Medicine Denizli Türkiye; ^35^ Department of Medical Oncology Kocaeli University Faculty of Medicine Kocaeli Türkiye; ^36^ Department of Medical Oncology Bursa City Hospital Bursa Türkiye; ^37^ Department of Medical Oncology Aksaray Training and Research Hospital Aksaray Türkiye; ^38^ Department of Medical Oncology Kütahya University of Health Sciences Kütahya Türkiye; ^39^ Department of Medical Oncology Demiroğlu Bilim University, İstanbul Florence Nightingale Hospital İstanbul Türkiye; ^40^ Department of Medical Oncology, Faculty of Medicine Sakarya University Sakarya Türkiye; ^41^ Department of Medical Oncology Antalya City Hospital Antalya Türkiye; ^42^ Department of Medical Oncology, Faculty of Medicine Muğla Sıtkı Koçman University Muğla Türkiye; ^43^ Department of Medical Oncology Başkent University Faculty of Medicine Adana Türkiye; ^44^ Fenerbahçe University, Vocational School of Health Services, Medical Oncology İstanbul Türkiye; ^45^ Department of Medical Oncology Bahçeşehir University Medicalpark Göztepe Hospital İstanbul Türkiye; ^46^ Department of Biomedical Sciences Humanitas University Milan Italy; ^47^ Humanitas Cancer Center IRCCS Humanitas Research Hospital Milan Italy

**Keywords:** adjuvant chemotherapy, biliary tract cancer, capecitabine, cholangiocarcinoma, gallbladder cancer

## Abstract

The recommended adjuvant chemotherapy (adj‐ChT) regimen for resected biliary tract cancers (BTC) is capecitabine (Cape); yet, the recommendation is based on limited evidence. Although varied adj‐ChTs have been employed in practice, robust real‐world data is scarce. We conducted a national, multicenter, hospital‐based registry study to evaluate adj‐ChTs in resected BTCs. Patients who received adj‐ChT (± radiotherapy) between 2010 and 2024 were included. Recurrence‐free (RFS) and overall survival (OS) were analyzed by adjusted Cox‐regression and propensity score‐based inverse‐probability‐of‐treatment‐weighting (IPTW), addressing selection bias. Among 617 patients from 44 centers, 513 were eligible. The most frequent adj‐ChTs were Cape (35.5% [*n* = 182]), gemcitabine–cisplatin (Gem‐Cis; 22.4% [*n* = 115]), gemcitabine–capecitabine (Gem‐Cape; 20.1% [*n* = 103]), and gemcitabine (Gem; 10.7% [*n* = 55]). Median RFS and OS with Cape were 19.7 (95% Confidence Interval [95% CI]: 14.2–41.1) and 41.9 months (95% CI: 25.9–69.2). In adjusted/controlled comparisons with Cape, no differences in RFS or OS were observed with Gem‐Cis (RFS: Hazard Ratio [HR] 1.13 [95% CI: 0.77–1.66]; OS: HR: 1.03 [95% CI: 0.66–1.61]), Gem‐Cape (RFS: HR 0.97 [95% CI: 0.68–1.38]; OS: HR: 0.81 [95% CI: 0.52–1.24]), or Gem (RFS: HR 1.00 [95% CI: 0.63–1.59]; OS: HR: 0.93 [95% CI: 0.55–1.57]). Similarly, IPTW analyses showed no difference in RFS and OS. Radiotherapy appeared to be associated with improved survival. Performance status, T‐stage, lymph‐node positivity, and R1‐resection were independently associated with RFS and OS. In conclusion, this real‐world study did not identify a regimen superior to Cape. Given the modest benefit of adj‐ChTs, novel approaches, including neoadjuvant and targeted/immunotherapy strategies, are needed.

## Abstract (highlights)

What's new?

The current recommended adjuvant chemotherapy regimen for resected biliary tract cancers is capecitabine. Although varied adjuvant chemotherapy regimens are also employed in practice, robust real‐world comparative data is scarce. This retrospective multicenter national study in a large real‐world cohort did not identify an adjuvant chemotherapy regimen superior to capecitabine in efficacy. The study confirmed the previously reported prognostic factors in resected biliary tract cancers. Analyses also showed a signal for a possible benefit of additional radiotherapy. Given the modest benefit of adjuvant chemotherapy regimens observed in this study, novel approaches, including neoadjuvant and targeted/immunotherapy strategies, are needed in this setting.

## Graphical abstract

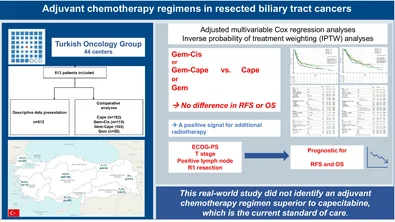

**Abbreviations**
Ajd‐ChT: adjuvant chemotherapy
BTC: biliary tract cancer
Cape: capecitabine
CCA: cholangiocarcinoma
CI: confidence interval
Cis‐5FU: cisplatin‐5fluouracil
CRT: chemoradiotherapy
ECOG‐PS: eastern cooperative oncology group performance score
ESMO: European Society of Medical Oncology
FOLFOX: folinic acid, fluorouracil, and oxaliplatin
Gem: gemcitabine
Gem‐Cape: gemcitabine‐capecitabine
Gem‐Carbo: gemcitabin‐carboplatin
Gem‐Cis: gemcitabine‐cisplatin
Gem‐OX: gemcitabine‐oxaliplatin
GI: gastrointestinal
HR: hazard ratio
IPTW: inverse‐probability‐of‐treatment‐weighting
LVI: lymphovascular invasion
OS: overall survival
PNI: perineural invasion
RFS: recurrence‐free survival
RT: radiotherapy
SMD: standardized mean difference
TOG: Turkish Oncology Group
XELOX: oxaliplatin‐capecitabin

## 1 Introduction

Biliary tract cancers (BTCs), encompassing intrahepatic, perihilar, distal cholangiocarcinomas (CCAs), and gallbladder cancers, are uncommon and account for approximately 3% of all gastrointestinal malignancies; however, their incidence is increasing [1]. Surgical resection remains the only potentially curative option; however, nearly 25% of patients are eligible for surgery at the time of diagnosis [2], and up to 70% of patients who undergo surgery eventually have recurrence [1].

Over the past decade, a limited number of randomized controlled trials (RCTs) have evaluated adjuvant chemotherapy (adj‐ChT) in patients with resected BTCs [3]. The BILCAP trial, which compared capecitabine (Cape) monotherapy to observation, did not meet its primary endpoint of overall survival (OS) in the intention‐to‐treat analysis; however, prespecified per‐protocol analysis demonstrated a statistically significant survival benefit [4, 5]. Consequently, Cape has been adopted as the standard adj‐ChT in Western countries and is recommended in the European Society for Medical Oncology (ESMO) guidelines with a category II/A level of evidence [1]. Nevertheless, the robustness of the evidence supporting this recommendation remains a topic of ongoing debate [6, 7]. The ASCOT trial conducted in Japan assessed the efficacy of S‐1 compared to observation and reported a significant OS benefit, supporting the benefit of fluoropyrimidines [8].

Conversely, the PRODIGE‐12 and BCAT trials failed to demonstrate improvements in either recurrence‐free survival (RFS) or OS with gemcitabine monotherapy (Gem) and the gemcitabine–oxaliplatin (Gem‐OX) combination, compared to observation [9, 10]. Given the established role of gemcitabine–cisplatin (Gem‐Cis) in the metastatic setting, a Phase II trial in Korea explored this regimen in the adjuvant setting for patients with node‐positive extrahepatic CCA compared to Cape but found no superiority [11].

Adjuvant therapy for BTCs remains an area of ongoing research, with current evidence offering limited guidance. A variety of chemotherapy regimens have been employed in routine clinical practice before and after the BILCAP trial. However, comprehensive real‐world data comparing the effectiveness of these regimens is lacking. This multicenter study was therefore conducted to evaluate the real‐world efficacy of different adj‐ChT regimens in patients with resected BTCs.

## 2 Methods

The detailed methodology of the study, including “Study design and oversight,” “Eligibility of patients,” “Data collection,” “Data quality management,” “Outcomes,” and “Statistical analyses,” STROBE and ESMO‐GROW checklists (Appendix 1 and 2), was presented in the Supporting Information.

Briefly, the SAFRADJU‐2501 study, initiated by the Turkish Oncology Group/Gastrointestinal Cancer Working Subgroup (TOG/GI), was designed as a nationwide, multicenter, hospital‐based, retrospective, analytical real‐world data registry. Patients aged 18 years or older, with histologically confirmed and surgically resected BTC, including intrahepatic, perihilar, distal CCA, and gallbladder cancer, were eligible for inclusion. Exclusion criteria included histological diagnoses other than adenocarcinoma, ampullary carcinoma, R2 surgical resection, absence of adj‐ChT, receipt of adjuvant RT or chemoradiotherapy (CRT) alone without adj‐ChT, and administration of neoadjuvant therapy before surgery.

The primary endpoints of the study were RFS and OS. The four most common adj‐ChT regimens were assessed by comparative analyses (Gem‐Cis, Gem‐Cape, Gem; vs. Cape). Regimens with a small number of patients were presented descriptively only. Missing data were handled by multiple imputation. To address prognostic factor differences and selection bias between adj‐ChT regimens, multivariable Cox regression analyses were performed, adjusting for age, sex, era, performance score (ECOG‐PS), primary tumor location, T stage, lymph node status, resection margin (R0 vs. R1), tumor grade, presence of lymphovascular invasion (LVI), perineural invasion (PNI), and receipt of adjuvant RT or CRT. Propensity scoring by inverse probability of treatment weighting (IPTW) was performed (Gem‐Cis vs. Cape, Gem‐Cape vs. Cape, Gem vs. Cape) to address selection bias further. Covariate balance between treatment groups before and after weighting was assessed using standardized mean differences (SMDs). Secondary endpoints included prespecified subgroup analyses and 2‐year RFS and OS of the four most common adj‐ChT regimens.

## 3 Results

### 3.1 Patients and Characteristics of the Study Population

Out of 617 patients with resected BTC identified from 44 hospitals with oncology units, 513 met the eligibility criteria and were included, covering cases diagnosed between 2010 and 2024 (Figure S1). 14.2% (*n* = 73) of patients were diagnosed between 2010 and 2015, 30.2% (*n* = 155) between 2015 and 2020, and 55.6% (*n* = 285) between 2020 and 2024. Patients were recruited from all seven geographical regions of Türkiye, with Central Anatolia being the most represented (*n* = 160, 31.2%) (Figure S2). Baseline demographic and clinicopathologic characteristics of the entire study population are summarized in Table 1. The median age was 62 years (range: 26–84), and 48.5% of patients (*n* = 249) were female. An ECOG‐PS of 0 was recorded in 30.8% of patients (*n* = 158). The most frequent tumor sites were the gallbladder (*n* = 218, 42.5%) and intrahepatic localization (*n* = 107, 20.9%). Positive lymph node involvement was observed in 32.9% of patients (*n* = 169), and 27.9% (*n* = 143) underwent R1 resections.

**TABLE 1:** Characteristics of study population (*n* = 513).

Age (median, min–max)	62 (26–84)
*Gender, n (%)*	
Male	264 (51.5)
Female	249 (48.5)
*Era, n (%)*	
2010–2015	73 (14.2)
2015–2020	155 (30.2)
2020–2024	285 (55.6)
*Eastern Cooperation Oncology Group (ECOG) performance score, n (%)*	
0	158 (30.8)
1	295 (57.5)
2	50 (9.8)
Unknown	10 (1.9)
*Localization, n (%)*	
Intrahepatic	107 (20.9)
Perihilar	70 (13.6)
Distal	91 (17.7)
Gallbladder	218 (42.5)
Not determined due to invasion	27 (5.3)
*T stage, n (%)*	
1	59 (11.5)
2	226 (44.1)
3	176 (34.3)
4	34 (6.6)
Not determined	18 (3.5)
*Lymph node status, n (%)*	
Positive	169 (32.9)
Negative	250 (48.7)
Unknown	94 (18.4)
*Resection, n (%)*	
R0	360 (70.2)
R1	143 (27.9)
Not reported	10 (1.9)
*Grade, n (%)*	
1	86 (16.8)
2	247 (48.1)
3	74 (14.4)
Unknown	106 (20.7)
*Lymphovascular invasion, n (%)*	
Present	253 (49.3)
Absent	141 (27.5)
Not reported	119 (23.2)
*Perineural invasion, n (%)*	
Present	255 (49.7)
Absent	127 (24.8)
Not reported	131 (25.6)

Details of adjuvant treatments and patient compliance are provided in Table 2. The most commonly administered adj‐ChT regimens were Cape (*n* = 182, 35.5%), Gem‐Cis (*n* = 115, 22.4%), gemcitabine‐capecitabine (Gem‐Cape; *n* = 103, 20.1%), and Gem (*n* = 55, 10.7%). Other regimens that were used uncommonly in the adjuvant setting were Gem‐OX (*n* = 16, 3.1%), folinic acid, fluorouracil, and oxaliplatin (FOLFOX) (*n* = 12, %2.3), oxaliplatin‐capecitabine (XELOX) (*n* = 14, 2.7%), gemcitabine‐carboplatin (*n* = 10, 1.9%) and cisplatin‐5‐FU (*n* = 6, 1.2%). Dose reductions were required in 19.3% (*n* = 99) of patients, and 75.6% (*n* = 388) completed all planned chemotherapy cycles. Completion rate did not differ among regimens (Table S1). In addition to chemotherapy, 26.1% (*n* = 134) received RT, and 11.3% (*n* = 58) received CRT. Of those, 82.7% of CRT (*n* = 48) were with Cape, 12.1% (*n* = 7) were with Gem, and 5.2% (*n* = 3) were with 5‐FU. CRT with Cape or 5‐FU followed the fluopyrimidine‐based adjuvant chemotherapies, and CRT with Gem followed the Gem‐based adjuvant chemotherapies. Radiotherapy (RT or CRT) receipt was associated with younger age, R1 resection, lymph node, and PNI positivity (Table S2). Patients who received CRT had less intrahepatic localization and more lymph node positivity than those who received RT (Table S3). The median follow‐up duration for the whole cohort was 32.9 months (95% CI: 25.8–40.0).

**TABLE 2:** Adjuvant treatments and compliance with chemotherapy (*n* = 513).

*Adjuvant chemotherapy regimen, n (%)*	
Capecitabine	182 (35.5)
Gemcitabine‐Cisplatin	115 (22.4)
Gemcitabine‐Capecitabine	103 (20.1)
Gemcitabine	55 (10.7)
Gem‐OX	16 (3.1)
XELOX	14 (2.7)
FOLFOX	12 (2.3)
Gemcitabine‐Carboplatin	10 (1.9)
Cisplatin‐5FU	6 (1.2)
*Dose reduction needed, n (%)*	
Yes	99 (19.3)
No	414 (80.7)
*Completed planned cycles, n (%)*	
Yes	388 (75.6)
No	125 (24.4)
*Additional adjuvant radiation*	
No	321 (62.6)
Radiotherapy alone	134 (26.1)
Chemoradiotherapy	58 (11.3)

Abbreviations: Cape, Capecitabine; Cis‐5FU, cisplatin‐5fluouracil; FOLFOX, folinic acid, fluorouracil, and oxaliplatin; Gem‐OX, Gemcitabin‐Oxaliplatin; XELOX, oxaliplatin‐capecitabine.

Baseline characteristics of Gem‐Cis, Gem‐Cape, and Gem groups were compared to the Cape (Table 3). The Gem‐Cis group had younger patients (*p* < 0.001), a higher percentage of T3 and T4 disease (*p* < 0.001), lymph node positivity (*p* = 0.002), grade 3 disease (*p* = 0.017), LVI (*p* = 0.001), and PNI (*p* = 0.012) than the Cape group. The Gem‐Cape group had younger patients (*p* = 0.012), a higher percentage of lymph node positivity (*p* = 0.018), R1 resection (*p* = 0.16), LVI (*p* = 0.040), and PNI (*p* < 0.001) than the Cape group and received more RT and CRT (*p* = 0.008). The Gem group had similar characteristics to the Cape group, except less intrahepatic localization (*p* = 0.027) and less lymph node positivity (*p* = 0.046) (Table 3).

**TABLE 3:** Baseline prognostic characteristics of the adjuvant chemotherapy groups compared to capecitabine.

	Cape (*n* = 182)	Gem‐Cis (*n* = 115)	*p* *	Gem‐Cape (*n* = 103)	*p* *	Gem (*n* = 55)	*p* *
Age (median, min‐max)	64 (29–84)	60 (26–75)	**< 0.001**	61 (38–83)	**0.012**	65 (39–83)	0.975
*Gender, n (%)*							
Male	91 (50)	52 (45.2)	0.422	61 (59.2)	0.134	27 (49.1)	0.906
Female	91 (50)	63 (54.8)		42 (40.8)		28 (50.9)	
*ECOG‐PS, n (%)*							
0	68 (37.4)	31 (27.0)	0.167	29 (28.2)	0.162	16 (29.1)	0.177
1	92 (50.5)	70 (60.9)		62 (60.2)		31 (56.4)	
2	15 (8.2)	12 (10.4)		11 (10.7)		8 (14.5)	
Unknown	7 (3.9)	2 (1.7)		1 (0.9)		0 (0)	
*Localization, n (%)*							
Intrahepatic	41 (22.6)	30 (26.0)	0.747	19 (18.5)	0.145	5 (9.1)	**0.027**
Perihilar	27 (14.8)	11 (9.6)		16 (15.5)		10 (18.2)	
Distal	26 (14.3)	17 (14.8)		27 (26.2)		16 (29.1)	
Gallbladder	77 (42.3)	50 (43.5)		37 (35.9)		23 (41.8)	
Not determined	11 (6.0)	7 (6.1)		4 (3.9)		1 (1.8)	
*T stage, n (%)*							
1	27 (14.8)	6 (5.2)	**< 0.001**	10 (9.7)	0.470	6 (10.8)	0.385
2	90 (49.5)	42 (36.5)		47 (45.6)		21 (38.2)	
3	52 (28.6)	46 (40.0)		38 (36.9)		22 (40.0)	
4	6 (3.3)	17 (14.8)		5 (4.9)		3 (5.5)	
Not determined	7 (3.8)	4 (3.5)		3 (2.9)		3 (5.5)	
*Lymph node status, n (%)*							
Positive	47 (25.8)	53 (46.1)	**0.002**	43 (41.8)	**0.018**	11 (20.0)	**0.046**
Negative	93 (51.1)	43 (37.4)		44 (42.7)		38 (69.1)	
Unknown	42 (23.1)	19 (16.5)		16 (15.5)		6 (10.9)	
*Resection, n (%)*							
R0	136 (74.7)	78 (67.8)	0.433	62 (60.2)	**0.016**	40 (72.8)	0.830
R1	42 (23.1)	34 (29.6)		40 (38.8)		13 (23.6)	
Not reported	4 (2.2)	3 (2.6)		1 (1.0)		2 (3.6)	
*Grade, n (%)*							
1	34 (18.7)	12 (10.5)	**0.017**	21 (20.4)	0.370	7 (12.7)	0.453
2	92 (50.5)	48 (41.7)		53 (51.5)		26 (47.3)	
3	20 (11.0)	23 (20.0)		16 (15.5)		6 (10.9)	
Unknown	36 (19.8)	32 (27.8)		13 (12.6)		16 (29.1)	
*Lymphovascular invasion, n (%)*							
Present	85 (46.7)	59 (51.3)	**0.001**	55 (53.4)	**0.040**	27 (49.1)	0.546
Absent	66 (36.3)	21 (18.3)		23 (22.3)		16 (29.1)	
Not reported	31 (17.0)	35 (30.4)		25 (24.3)		12 (21.8)	
*Perineural invasion, n (%)*							
Present	82 (45.1)	52 (45.2)	**0.012**	67 (65.1)	**< 0.001**	28 (50.9)	0.338
Absent	62 (34.1)	24 (20.9)		13 (12.6)		13 (23.6)	
Not reported	38 (20.8)	39 (33.9)		23 (22.3)		14 (25.5)	
*Additional adjuvant radiation*							
No	121 (66.5)	74 (64.3)	0.515	49 (47.6)	**0.008**	36 (65.5)	0.536
Radiotherapy alone	40 (22.0)	31 (27.0)		35 (34.0)		15 (27.3)	
Chemoradiotherapy	21 (11.5)	10 (8.7)		19 (18.4)		4 (7.2)	

*Note:* The values written in bold reflects statistically significance (*p* < 0.05).

Abbreviations: Cape, Capecitabine; ECOG PS, European Cooperation Oncology Group performance score; Gem, Gemcitabine; Gem‐Cape, Gemcitabine‐capecitabine; Gem‐Cis, Gemcitabin‐cisplatin; IPTW, inverse probability of treatment weighting.

* Comparisons to Cape.

### 3.2 Recurrence‐Free Survival

A total of 307 events (59.8%) were recorded among the 513 patients during the follow‐up period. The median RFS for the entire cohort was 15.4 months (95% CI: 13.6–18.8). Kaplan–Meier survival curves and median RFS estimates for all regimens, including both common and uncommon regimens, are presented in Figure S3. Median RFS for patients receiving Cape, Gem‐Cis, Gem‐Cape, and Gem alone were 19.7 months (95% CI: 14.2–41.1), 12.2 months (95% CI: 10.7–15.2), 18.2 months (95% CI: 13.2–23.3), and 20.0 months (95% CI: 13.9–41.8), respectively (Figure 1A). (Univariable/unadjusted comparisons with Cape; Gem‐Cis: HR: 1.41 [95% CI: 1.04–1.92], Gem‐Cape: HR: 1.17 [95% CI: 0.85–1.61], Gem: HR: 0.97 [95% CI: 0.68–1.43]).

**FIGURE 1:**
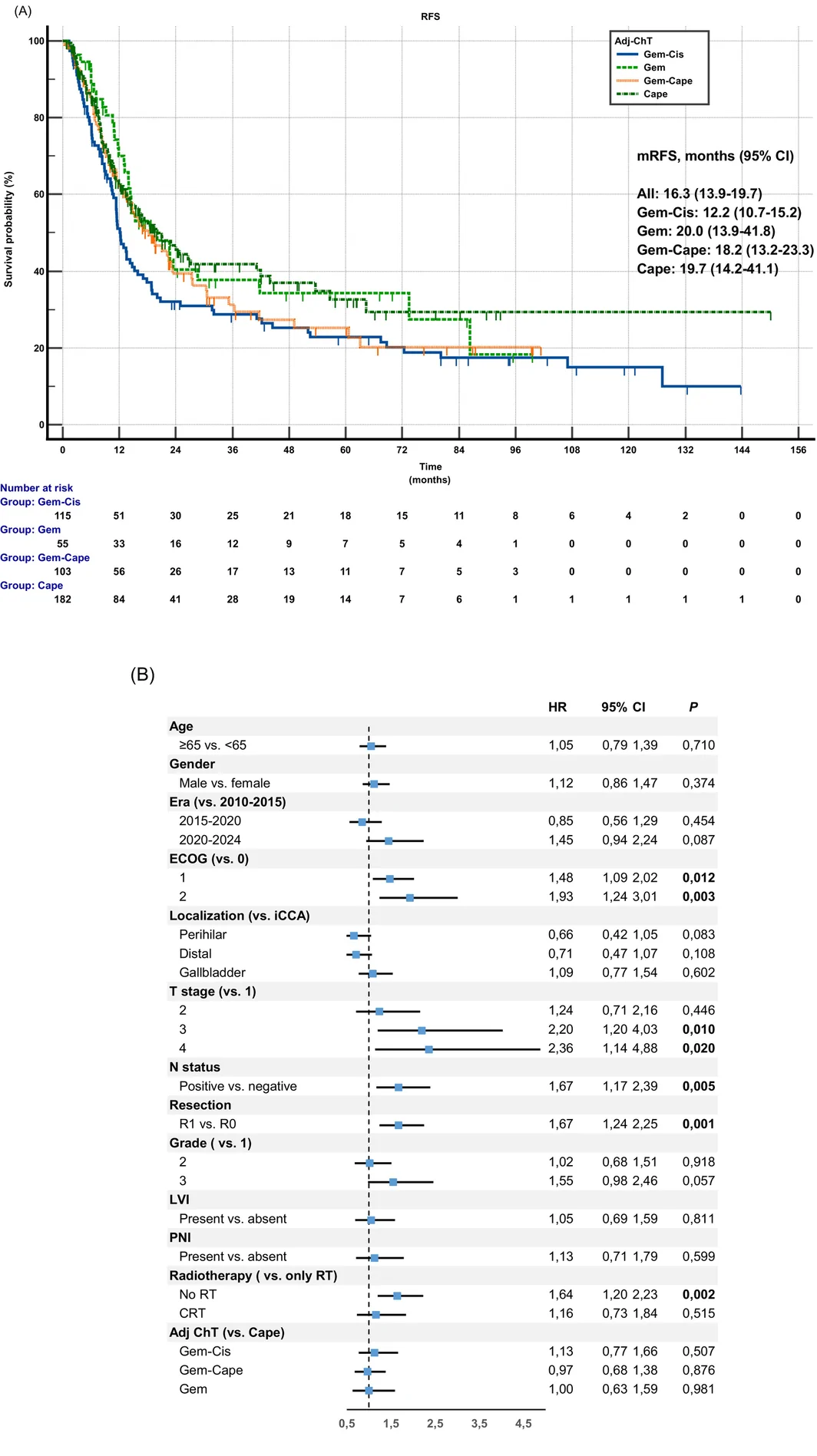
(A) Recurrence‐free survival (RFS) of Cape, Gem‐Cis, Gem‐Cape, and Gem: Kaplan–Meier plots and median RFS in months. (B) Adjusted analysis: Forest plot presentation of multivariable Cox regression analysis for RFS. Global (joint) *p* values: Era: 0.084, Localization: 0.033, T‐stage: < 0.001, Grade: 0.042, ECOG‐PS: 0.004, Adj‐ChT: 0.844, Radiotherapy: 0.004. Adj ChT, Adjuvant chemotherapy; Cape, Capecitabine; CI, Confidence interval; CRT, Chemoradiotherapy; ECOG, Eastern Cooperative Oncology Group performance score; Gem, Gemcitabine; Gem‐Cape, Gemcitabine‐capecitabine; Gem‐Cis, Gemcitabin‐cisplatin; HR, Hazard ratio; LVI, Lymhovascular invasion; mo, Months; mRFS, Median RFS; PNI, Perineural invasion; RT, Radiotherapy.

After adjustment for baseline clinical and pathological variables in multivariable Cox regression analysis, Gem‐Cis, Gem‐Cape, and Gem did not show statistically significant differences from Cape (Figure 1B). As a note, receipt of additional RT was independently associated with improved RFS (No RT vs. RT: HR 1.64; 95% CI: 1.20–2.23; *p* = 0.002), whereas CRT did not differ from RT. Additionally, multivariable Cox regression analysis identified several independent predictors of poorer RFS (Figure 1B), including ECOG‐PS, T‐stage, lymph node positivity, and R1 resection status.

### 3.3 Overall Survival

During the follow‐up period, 233 of 513 patients (45.4%) died. The median OS for the study population was 32.9 months (95% CI: 28.6–42.6). Kaplan–Meier survival curves and median OS estimates for all regimens, including both common and uncommon regimens, are presented in Figure S4. Median OS for patients treated with Cape, Gem‐Cis, Gem‐Cape, and Gem monotherapy were 41.9 months (95% CI: 25.9–69.2), 26.7 months (95% CI: 21.6–42.6), 41.3 months (95% CI: 27.8–61.1), and 43.9 months (95% CI: 29.3–70.2), respectively (Figure 2A). [Univariable/unadjusted comparisons with Cape; Gem‐Cis: HR: 1.23 (95% CI: 0.86–1.76), Gem‐Cape: HR: 0.97 (95% CI: 0.68–1.43), Gem: HR: 0.97 (95% CI: 0.63–1.51)].

**FIGURE 2:**
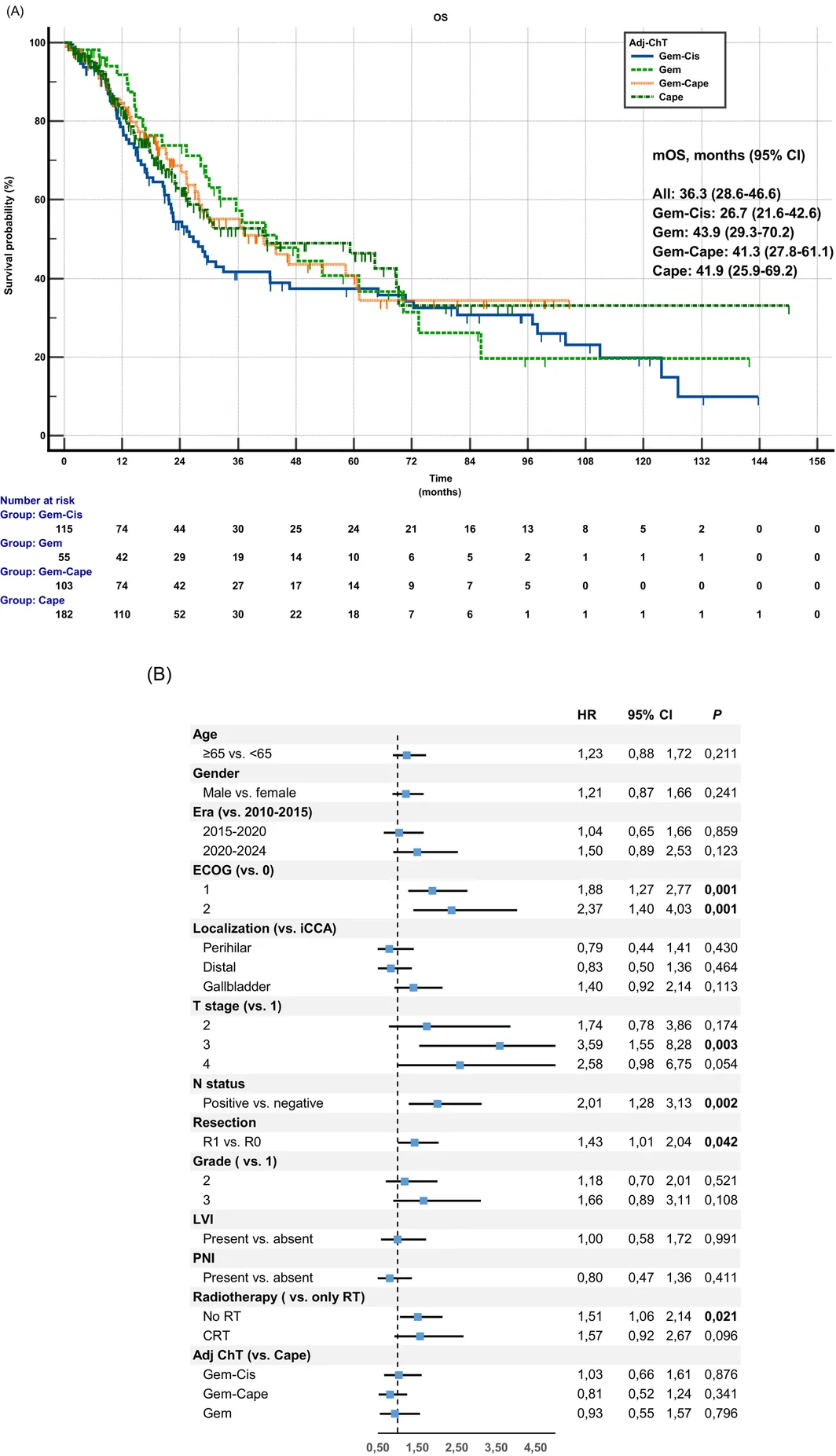
(A) Overall survival (OS) of Cape, Gem‐Cis, Gem‐Cape, and Gem: Kaplan–Meier plots and median OS in months. (B) Adjusted analysis: Forest plot presentation of multivariable Cox regression analysis for OS. Global (joint) *p* values: Era: 0.117, Localization: 0.032, T‐stage: < 0.001, Grade: 0.109, ECOG‐PS: 0.001, Adj‐ChT: 0.665, Radiotherapy: 0.039. Adj ChT, Adjuvant chemotherapy; Cape, Capecitabine; CI, Confidence interval; CRT, Chemoradiotherapy; ECOG, Eastern Cooperative Oncology Group performance score; Gem, Gemcitabine; Gem‐Cape, Gemcitabine‐capecitabine; Gem‐Cis, Gemcitabin‐cisplatin; HR, Hazard ratio; LVI, Lymhovascular invasion; mo, Months; mOS, Median OS; PNI, Perineural invasion; RT, Radiotherapy.

After adjustment for baseline clinical and pathological variables in multivariable Cox regression analysis, no significant differences were observed for Gem‐Cis, Gem‐Cape, and Gem compared to Cape (Figure 2B). Receipt of additional RT was independently associated with improved OS (No RT vs. RT: HR 1.51; 95% CI: 1.06–2.14; *p* = 0.021), whereas CRT did not differ from RT. Additionally, multivariable Cox regression analysis identified independent predictors of worse OS (Figure 2B), including ECOG‐PS, T stage, lymph node positivity, and R1 resection.

### 3.4 Inverse Probability of Treatment Weighting (IPTW)

IPTW analyses were performed for Gem‐Cis versus Cape, Gem‐Cape versus Cape, and Gem versus Cape. Covariate balance was evaluated using SMD and presented via Love plots (Figures S5A–C). In IPTW analyses, the RFS and OS with Gem‐Cis, Gem‐Cape, and Gem did not differ when compared to Cape. Since the era and T‐stage variables for Gem‐Cis versus Cape, and the era variable for Gem versus Cape, showed SMDs between 0.1 and 0.2 (Figure S5), additional double adjustments were performed. The results showed no difference between adj‐ChT regimens and Cape after the adjustment. The HRs and 95% CI of comparisons in IPTW were presented in Table 4.

**TABLE 4:** Recurrence‐free survival (RFS) and overall survival (OS) with adjuvant chemotherapies compared to Capecitabine after propensity scoring by inverse probability of treatment weighting (IPTW).

	Gem‐Cis vs. Cape^a^		Gem‐Cape vs. Cape		Gem vs. Cape^b^	
	HR (95% CI)	*p*	HR (95% CI)	*p*	HR (95% CI)	*p*
RFS	1.17 (0.49–2.75)	0.712	0.96 (0.41–2.23)	0.928	0.97 (0.35–2.69)	0.962
OS	0.92 (0.36–2.34)	0.875	0.79 (0.31–2.01)	0.634	0.85 (0.30–2.39)	0.758

Abbreviations: Cape, Capecitabine; CI, confidence interval; Gem, Gemcitabine; Gem‐Cape, Gemcitabine‐capecitabine; Gem‐Cis, Gemcitabin‐cisplatin; HR, Hazard ratio; OS, overall survival; RFS, recurrence‐free survival.

^a^ Double adjustment for era and T (as the SMDs were between 0.1 and 0.2) was performed. RFS: 1.12 (0.44–2.83), *p* = 0.805, OS: 0.85 (0.30–2.40), *p* = 0.768.

^b^ Double adjustment for era (as the SMD was between 0.1 and 0.2) was performed. RFS: 0.98 (0.33–2.88), *p* = 0.938, OS: 0.80 (0.26–2.41), *p* = 694.

### 3.5 2‐Year Survivals

The 2‐year RMST for RFS and OS for the Cape, Gem‐Cis, Gem‐Cape, and Gem regimens were presented in Table S4. 2‐year RFS and OS for Cape were 16.4 months (95% CI: 15.1–17.7) and 19.9 months (95% CI: 18.8–20.9), respectively. Comparisons (univariable, unadjusted) were presented in Table S5.

### 3.6 Subgroup Analyses

Prespecified multivariable‐adjusted subgroup analyses stratified by lymph node status, resection status, and anatomical localization are presented in Figures S6A–C. Overall, adjusted comparisons did not reveal a consistent survival advantage for any specific adj‐ChT regimen within the examined subgroups. However, a hypothesis‐generating/exploratory OS difference was observed for Gem‐Cis compared to Cape in patients with distal CCA (*p* = 0.036), as shown in Figure S6C.

## 4 Discussion

This multicenter real‐world study on adj‐ChT regimens for resected BTCs presented insights from multiple perspectives, including the landscape of the real‐world patient population, treatment patterns, efficacy of various regimens, and prognostic factors.

Cape remains the only adj‐ChT regimen recommended in Western guidelines, based on the BILCAP trial, which supports its use with a category II/A level of evidence according to the ESMO guidelines for BTCs and as category 1 in NCCN guidelines [1]. However, the strength of this recommendation remains debated, as the BILCAP trial did not demonstrate a statistically significant benefit in OS in the intention‐to‐treat population [6, 7]. This uncertainty, in addition to patients who were treated before the BILCAP trial and the Cape recommendation, may have contributed to the heterogeneous use of alternative adjuvant regimens in the routine clinical practice of our study population, such as Gem‐Cis or Gem‐Cape, which are not included in the guidelines and which may have been extrapolated from the metastatic setting of CCA or pancreatic cancer. Indeed, Cape is not completely implemented globally, and Gem‐Cis is still used as an adjuvant, for example, in India. An additional possible factor is patient comorbidities that may preclude a specific regimen.

Despite its established role in the metastatic setting, Gem‐Cis failed to demonstrate superiority over Cape in the adjuvant setting in the Phase II STAMP trial, in Asian patients with node‐positive extrahepatic CCA [11], a finding echoed by our results. In our study, patients in the Gem‐Cis and Gem‐Cape groups had worse prognostic factors, suggesting that these regimens may be options in high‐risk patients. The ongoing Phase III ACTICCA‐1 trial (NCT02170090) aims to further elucidate the role of Gem‐Cis versus Cape in the adjuvant setting. Similarly, Gem‐Cape is being evaluated in two ongoing randomized Phase III trials, BilGemCap (NCT0005056) [12] and AdBTC‐1 (NCT03779035), to determine its efficacy compared to Cape.

Previous trials have also failed to demonstrate a survival benefit with other regimens. In the BCAT study, Gem monotherapy did not improve outcomes compared to observation [9]. Likewise, the PRODIGE‐12 trial reported no benefit of Gem‐OX over observation [10], with early analyses suggesting a potential detrimental effect on 2‐year outcomes [13]. Our study included the descriptive data of uncommon regimens such as FOLFOX and XELOX, showing survivals that were not promising; however, the small number of patients and potential selection bias preclude drawing conclusions for these regimens in our study.

Although Cape is considered the standard adjuvant treatment based on the BILCAP trial, real‐world data supporting its effectiveness have been limited. A notable example is a French real‐world cohort presented as a conference abstract, which included 324 patients, 200 treated with Cape and 124 under observation [14]. Using inverse probability of treatment weighting (IPTW), the study demonstrated a statistically significant improvement in DFS for the Cape group compared to observation (IPTW HR: 0.76; 95% CI: 0.60–0.96; *p* = 0.0224), although no significant difference was observed in OS. In this cohort, median DFS and OS with Cape were 15.8 and 43.5 months, respectively. Consistent with these findings, our real‐world study showed a median RFS of 19.7 months and OS of 41.9 months with Cape. In contrast, the BILCAP trial reported longer median RFS and OS durations of 24.3 and 52.3 months, respectively [4]. These discrepancies may highlight the potential vulnerability of patients with BTCs in routine clinical practice and suggest that outcomes observed in clinical trials may not fully reflect the broader real‐world patient population. Indeed, a significant proportion of patients with BTC are not eligible or intolerant to chemotherapy in the real world; however, the number of adj‐ChT cycles may be associated with OS [15].

Our study reaffirmed the prognostic significance of several clinicopathological factors previously reported in the literature. Exploratory analyses from the BILCAP trial identified R1 resection margins, nodal involvement, and poor tumor differentiation as predictors of worse survival, although Cape did not confer a significant benefit within these high‐risk subgroups [4]. However, as the study was not powered to detect effects in subgroups, more research was recommended. In line with these findings, our multivariable analysis demonstrated that ECOG‐PS, advanced T stage, lymph node positivity, and R1 resection were independently associated with both RFS and OS. These results underscore the importance of integrating these prognostic markers into routine risk stratification.

The role of RT in the adjuvant treatment of BTCs remains uncertain due to the lack of prospective randomized trials comparing adjuvant sequential adj‐ChT and RT to adj‐ChT alone. The SWOG S0809 trial, a single‐arm Phase II study, evaluated the regimen of Gem‐Cape followed by concurrent CRT in patients with high‐risk resected extrahepatic CCA and gallbladder cancer [16]. However, the absence of a control arm limits the interpretability of the results. A retrospective analysis from the American College of Surgeons National Cancer Database indicated improved survival with adjuvant CRT compared to surgery alone in patients with eCCA [17]. Notably, neither study directly compared CRT to chemotherapy alone. In our cohort, the addition of RT to adj‐ChT was independently associated with improved RFS and OS compared to adj‐ChT alone. CRT did not differ from RT. However, due to the acknowledged limitations, further prospective studies are warranted to evaluate the optimal integration of RT in the adjuvant setting.

The results of this study suggest that the efficacy of various adj‐ChT regimens in resected BTCs remains limited in real‐world settings, as more than 50% of recurrences occur in the first 2 years of adj‐ChT. Even with Cape, the observed benefit appears to be attenuated in routine clinical practice compared to controlled clinical trial settings. Collectively, these results highlight an urgent need for a paradigm shift in the perioperative management of BTCs. Ongoing efforts are exploring novel approaches to improve outcomes. The Phase II ADJUBIL trial investigates adjuvant durvalumab and tremelimumab in combination with or without Cape. A recent report of the trial suggested no benefit from adding Cape to durvalumab‐tremelimumab [18]. The ARTEMIDE‐Biliary01 trial (NCT06109779) is currently evaluating adj‐ChT combined with the PD‐1/TIGIT bispecific antibody rilvegostomig. Additionally, the potential role of targeted therapies such as FGFR and IDH inhibitors in the adjuvant setting remains an important area for future research. A growing body of investigation is also focused on neoadjuvant strategies. Phase II single‐arm NEO‐GAP trial suggested the feasibility of neoadjuvant Gem‐Cis‐nab‐paclitaxel for resectable, high‐risk intrahepatic CCA [19]. ECOG‐ARCIN (NCT04559139) is evaluating perioperative Gem‐Cis with or without nab‐paclitaxel. The Phase 2/3 PURITY trial (NCT06037980) is investigating neoadjuvant Gem‐Cis‐Nabpaclitaxel, surgery, and adjuvant Cape compared to upfront surgery and adjuvant Cape. The DEBATE study (NCT04308174) is examining durvalumab in both adjuvant and perioperative settings alongside Gem‐Cis [3]. Furthermore, recent congress abstracts have reported the initial results of the GAIN study (perioperative Gem‐Cis) [20] and POLCAGB (neoadjuvant chemotherapy ± CRT) [21]. These ongoing studies reflect a future landscape that will likely incorporate neoadjuvant approaches, immunotherapy, and molecularly targeted agents into the treatment paradigm for resectable BTCs.

This study has several strengths. The BTCs are relatively rare; only 20% of patients have resectable disease, and two‐thirds of them are eligible to receive adjuvant ChT. Thus, to our knowledge, this study represents the largest real‐world cohort assessing adj‐ChT in resected BTCs and is the first to assess a broad range of chemotherapeutic regimens within a single analysis. Although the study was conducted within a single country, it included data from 44 centers distributed across diverse geographic and demographic regions. Given the genetic admixture of the population of Türkiye, encompassing both European and Asian ancestry, our cohort may reflect a more globally representative patient population. Additionally, the inclusion of data on RT and validation of established prognostic factors strengthens the comprehensiveness of the analysis.

Nevertheless, this study is subject to the inherent limitations of its retrospective design. The percentage of patients registered in time periods increased from 2010–2015 to 2020–2024, which may be explained by the accessibility of data. Our study had a prespecified maximum follow‐up duration as an exclusion criterion (for three patients); however, censoring at the maximum study duration would have been the methodologically more correct approach. Despite the implementation of data quality control strategies, variability in local pathological and radiological assessments that are not centralized, as well as variability in chemotherapy protocols, RT techniques, and the timing of tumor assessments, introduces heterogeneity. The definition of an R1 resection was unlikely to be standardized across 44 centers over 14 years, and the lack of standardization is an important source of heterogeneity. Our study did not include an observation group, since only patients who have a condition precluding adj‐ChT undergo observation in the real world. A longer follow‐up would be more informative. Even with IPTW, residual bias is difficult to eliminate in retrospective data. Multivariable and IPTW analyses were not performed due to the limited sample size in some of the adj‐ChT regimens, such as FOLFOX, and conclusions for these regimens require further research. The results regarding RT and CRT may be difficult to interpret without details on indication, target volumes, dose, or patient selection criteria. Furthermore, due to limitations in documentation and grading, toxicity data could not be reliably collected or analyzed.

## 5 Conclusion

This real‐world data does not warrant any adj‐ChT regimen (neither Gem‐Cis nor Gem‐Cape nor Gem) against Cape. While efficacy appears similar, no conclusions can be drawn about the comparative toxicity and risk–benefit ratio of the regimens. RT added to adj‐ChT may confer benefit; however, further investigation in trials with well‐defined methodology and design is required to set optimal use of RT. The prognostic roles of ECOG‐PS, T stage, lymph node positivity, and resection status were confirmed. However, these results were limited by the retrospective nature of the data. The modest benefit of various chemotherapy regimens emphasizes a need for a paradigm change in the adjuvant setting, such as a perioperative approach or novel therapies, including immunotherapy and targeted agents. Ongoing trials will shed light on the perioperative management of resected BTCs.

## Author Contributions

**Erman Akkus:** conceptualization, investigation, writing – original draft, writing – review and editing, visualization, methodology, formal analysis, data curation. **Mehmet Kayaalp:** conceptualization, data curation, writing – review and editing, methodology. **Müzeyyen Aslı Ergözoğlu:** conceptualization, data curation, writing – review and editing, methodology. **Ilgın Koç Kuş:** conceptualization, methodology, writing – review and editing, data curation. **Doğan Bayram:** conceptualization, data curation, methodology, writing – review and editing. **Bilgeşah Kılıçtaş:** conceptualization, methodology, writing – review and editing, data curation. **Emel Ayvaz Güneyin:** conceptualization, writing – review and editing, methodology, data curation. **Ali Kalem:** conceptualization, methodology, writing – review and editing, data curation. **Haydar Temizyürek:** conceptualization, methodology, data curation, writing – review and editing. **Mehmet Mutlu Çatlı:** conceptualization, methodology, writing – review and editing, data curation. **Mehmet Sinan Akarca:** conceptualization, writing – review and editing, methodology, data curation. **Maral Martin Mıldanoğlu:** conceptualization, writing – review and editing, methodology, data curation. **Görkem Turhan:** conceptualization, methodology, writing – review and editing, data curation. **Mahmut Kara:** conceptualization, methodology, writing – review and editing, data curation. **Hacı Arak:** conceptualization, writing – review and editing, methodology, data curation. **Feyza Arslan Tan:** conceptualization, methodology, writing – review and editing, data curation. **Burak Paçacı:** conceptualization, methodology, writing – review and editing, data curation. **Berkay Yeşilyurt:** conceptualization, methodology, writing – review and editing, data curation. **Orhun Akdoğan:** conceptualization, methodology, writing – review and editing, data curation. **Emine Bihter Çetin:** conceptualization, methodology, writing – review and editing, data curation. **Selahattin Çelik:** conceptualization, methodology, writing – review and editing, data curation. **Fatma Keskin Uzundere:** conceptualization, methodology, writing – review and editing, data curation. **Elif Şahin:** conceptualization, methodology, writing – review and editing, data curation. **Zehra Sadak Öcal:** conceptualization, methodology, writing – review and editing, data curation. **Lamia Şeker Can:** conceptualization, methodology, writing – review and editing, data curation. **Bahadır Köylü:** conceptualization, methodology, writing – review and editing, data curation. **Mehmet Cem Fidan:** conceptualization, methodology, writing – review and editing, data curation. **Esmanur Kaplan Tüzün:** conceptualization, methodology, writing – review and editing, data curation. **Teoman Şakalar:** conceptualization, methodology, writing – review and editing, data curation. **Elif Sertesen Çamöz:** conceptualization, methodology, writing – review and editing, data curation. **Haydar Çağatay Yüksel:** conceptualization, methodology, writing – review and editing, data curation. **Onur Yazdan Balçık:** conceptualization, methodology, writing – review and editing, data curation. **Pınar Peker:** conceptualization, methodology, writing – review and editing, data curation. **Mehmet Yılmaz:** conceptualization, methodology, writing – review and editing, data curation. **Şeymanur Ala Enli:** conceptualization, methodology, writing – review and editing, data curation. **Erdem Kölemen:** conceptualization, methodology, writing – review and editing, data curation. **Sibel Oyucu Orhan:** conceptualization, methodology, writing – review and editing, data curation. **Yakup Düzköprü:** conceptualization, methodology, writing – review and editing, data curation. **Mustafa Ersoy:** conceptualization, methodology, writing – review and editing, data curation. **Tuğba Akın Telli:** conceptualization, methodology, writing – review and editing, data curation. **Mesut Yılmaz:** conceptualization, methodology, writing – review and editing, data curation. **Yusuf İlhan:** conceptualization, methodology, writing – review and editing, data curation. **Atila Yıldırım:** conceptualization, methodology, writing – review and editing, data curation. **Ramazan Coşar:** conceptualization, methodology, writing – review and editing, data curation. **Müslih Ürün:** conceptualization, methodology, writing – review and editing, data curation. **Arif Hakan Önder:** conceptualization, methodology, writing – review and editing, data curation. **Murat Araz:** conceptualization, methodology, writing – review and editing, data curation. **Ertuğrul Bayram:** conceptualization, methodology, writing – review and editing, data curation. **Havva Yeşil Çınkır:** conceptualization, methodology, writing – review and editing, data curation. **Ali Alkan:** conceptualization, methodology, writing – review and editing, data curation. **Mustafa Gürbüz:** conceptualization, methodology, writing – review and editing, data curation. **Nilüfer Avcı:** conceptualization, methodology, writing – review and editing, data curation. **Fatma Paksoy Türköz:** conceptualization, methodology, writing – review and editing, data curation. **İsmail Oğuz Kara:** conceptualization, methodology, writing – review and editing, data curation. **Fatih Köse:** conceptualization, methodology, writing – review and editing, data curation. **İlkay Tuğba Ünek:** conceptualization, methodology, writing – review and editing, data curation. **Ahmet Bilici:** conceptualization, methodology, writing – review and editing, data curation. **Öznur Bal:** conceptualization, methodology, writing – review and editing, data curation. **Şuayib Yalçın:** conceptualization, methodology, writing – review and editing, data curation. **Lorenza Rimassa:** conceptualization, writing – review and editing, writing – original draft, methodology, supervision. **Hatime Arzu Yaşar:** conceptualization, methodology, formal analysis, investigation, writing – review and editing, writing – original draft, supervision, data curation.

## Ethics Statement

Ethical approval was obtained from the Clinical Research Ethics Committee of Ankara University Faculty of Medicine (Initial approval number: İ09‐714‐24 [01.11.2024], revision approval number: İ04‐342‐25 [05.05.2025]), in accordance with the Declaration of Helsinki and Good Clinical Practice (GCP) guidelines (ClinicalTrials.gov: NCT06975917).

## Conflicts of Interest

E.A. received a “Virtual Mobility Grant” from COST (European Cooperation in Science and Technology) based on COST action CA22125. L.R. reports Consulting/advisory role: AbbVie, AstraZeneca, Basilea, Bayer, BMS, Boehringer Ingelheim, Eisai, Elevar Therapeutics, Exelixis, Genenta, Guerbet, Hengrui, Incyte, Ipsen, Jazz Pharmaceuticals, MSD, Nerviano Medical Sciences, Roche, Servier, Taiho Oncology, Zymeworks Lecture fees: AstraZeneca, Bayer, Biologix, BMS, Eisai, Guerbet, Incyte, Ipsen, Roche, Servier Travel Expenses: AstraZeneca, Servier Research funding to her institution: AbbVie, AstraZeneca, BeiGene, BMS, Exelixis, Fibrogen, Incyte, Ipsen, Jazz Pharmaceuticals, MSD, Nerviano Medical Sciences, Roche, Servier, Taiho Oncology, TransThera Sciences, Zymeworks. The other authors declare no conflicts of interest.

## Supporting information


**Figure S1:** ESMO‐GROW flowchart for real‐world evidence studies in oncology: Formation and selection of the study population.
**Figure S2:** Distribution of patients (*n* = 513) across Türkiye. Türkiye bridges the continents of Europe and Asia as shown in the figure. It comprises seven geographical regions named on the map. The study population was distributed throughout these regions from Europe to Asia.
**Figure S3:** Recurrence‐free survivals (RFS) according to the adjuvant chemotherapy regimens: Kaplan–Meier plots and median RFS in months.
**Figure S4:** Overall survival (OS) according to the adjuvant chemotherapy regimens: Kaplan–Meier plots and median OS in months.
**Figure S5:** Love plot presentations of covariate balance evaluation by standardized mean difference (threshold < 0.1) after inverse probability of treatment weighting (IPTW). (A) Gem‐Cis vs. Cape, (B) Gem‐Cape vs. Cape, (C) Gem vs. Cape.
**Figure S6:** Prespecified adjusted subgroup analyses (a) RFS and OS for lymph node (N) negative and positive groups, (b) RFS and OS for R0 and R1 resection groups, (c) RFS and OS for anatomical subtypes.
**Table S1:** Completion rates between chemotherapy regimens.
**Table S2:** Comparison of characteristics between patients who received and did not receive radiotherapy (RT or CRT).
**Table S3:** Comparison of characteristics between patients who received CRT and RT.
**Table S4:** 2‐year restricted mean survival times (RMST) for recurrence‐free survival (RFS) and overall survival (OS).
**Table S5:** 2‐year restricted mean survival time comparison for RFS and OS (Capecitabine vs. other regimens)*.
**Appendix 1:** STROBE Statement—checklist of items that should be included in reports of observational studies.
**Appendix 2:** ESMO‐GROW checklist for authors and reviewers.

## Data Availability

The data that support the findings of this study are available from the corresponding author upon reasonable request.
