# Supplementary material for: Adjuvant Chemotherapy Regimens in Resected Biliary Tract Cancers: National, Comparative, Observational Study (TOG/GI‐SAFRADJU‐2501)
Source: Int J Cancer. 2026 Jun 29;159(11):2766–77. doi: 10.1002/ijc.70623 (PMC13637066; doi:10.1002/ijc.70623)
Supplement: Supplementary file 1 [file IJC-159-2766-s001.pdf]

Supplementary Materials and Methods

Adjuvant Chemotherapy Regimens in Resected Biliary Tract Cancers: National, Comparative, Observational Study (TOG/GI-SAFRADJU-2501)

Erman Akkus, Mehmet Kayaalp, Müzeyyen Aslı Ergözoğlu, Ilgın Koç Kuş, Doğan Bayram, Bilgeşah Kılıçtaş, Emel Ayvaz Güneyin, Ali Kalem, Haydar Temizyürek, Mehmet Mutlu Çatlı, Mehmet Sinan Akarca, Maral Martin Mıldanoğlu, Görkem Turhan, Mahmut Kara, Hacı Arak, Feyza Arslan Tan, Burak Paçacı, Berkay Yeşilyurt, Orhun Akdoğan, Emine Bihter Çetin, Selahattin Çelik, Fatma Keskin Uzundere, Elif Şahin, Zehra Sadak Öcal, Lamia Şeker Can, Bahadır Köylü, Mehmet Cem Fidan, Esmanur Kaplan Tüzün, Teoman Şakalar, Elif Sertesen Çamöz, Haydar Çağatay Yüksel, Onur Yazdan Balçık, Pınar Peker, Mehmet Yılmaz, Şeymanur Ala Enli, Erdem Kölemen, Sibel Oyucu Orhan, Yakup Düzköprü, Mustafa Ersoy, Tuğba Akın Telli, Mesut Yılmaz, Yusuf İlhan, Atila Yıldırım, Ramazan Coşar, Müslih Ürün, Arif Hakan Önder, Murat Araz, Ertuğrul Bayram, Havva Yeşil Çınkır, Ali Alkan, Mustafa Gürbüz, Nilüfer Avcı, Fatma Paksoy Türköz, İsmail Oğuz Kara, Fatih Köse, İlkay Tuğba Ünek, Ahmet Bilici, Öznur Bal, Şuayib Yalçın, Lorenza Rimassa, Hatime Arzu Yaşar

Table of Contents

1. Supplementary materials and methods
   1.1. Study design and oversight
   1.2. Eligibility of patients
   1.3. Data collection
   1.4. Data quality management
   1.5. Outcome
   1.6. Statistical analyses
2. Supplementary figures

   Figure S1. ESMO-GROW flowchart for real-world evidence studies in oncology: Formation and selection of the study population.

Figure S2. Distribution of patients (n=513) across Türkiye.

Figure S3. Recurrence-free survivals (RFS) according to the adjuvant chemotherapy regimens: Kaplan-Meier plots and median RFS in months.

Figure S4. Overall survival (OS) according to the adjuvant chemotherapy regimens: Kaplan-Meier plots and median OS in months.

Figure S5. Love plot presentations of covariate balance evaluation by standardized mean difference (threshold <0.1) after inverse probability of treatment weighting (IPTW). A) Gem-Cis vs. Cape, B) Gem-Cape vs Cape, C) Gem vs Cape.

Figure S6. Prespecified adjusted subgroup analyses a) RFS and OS for lymph node (N) negative and positive groups, b) RFS and OS for R0 and R1 resection groups, c) RFS and OS for anatomical subtypes.

3. Supplementary tables

   Table S1. Completion rates between chemotherapy regimens.

   Table S2. Comparison of characteristics between patients who received and did not receive radiotherapy (RT or CRT).

   Table S3. Comparison of characteristics between patients who received CRT and RT.

   Table S4. 2-year restricted mean survival times (RMST) for recurrence-free survival (RFS) and overall survival (OS).

   Table S5. 2-year restricted mean survival time comparison for RFS and OS (Capecitabine vs other regimens).

4. Supplementary references
5. Appendix 1. STROBE checklist
6. Appendix 2. ESMO-GROW checklist

1. Materials and Methods

1.1. Study design and oversight

The SAFRADJU-2501 study, initiated by the Turkish Oncology Group / Gastrointestinal Cancer Working Subgroup (TOG/GI), was designed as a nationwide, multicenter, hospital-based, retrospective, analytical real-world data registry study. The study protocol was presented and approved during the 2024 Annual Meeting of the Turkish Oncology Group (TOG). Oncology centers across Türkiye were invited to participate via an online announcement circulated through TOG communication channels. A standardized data collection template was provided to participating centers. Institutions unable to secure local ethical approval were excluded from the study. The protocol was registered at ClinicalTrials.gov (NCT06975917). Data collection was conducted over a nine-month period, from November 2024 to July 2025.

Ethical approval was obtained from the Clinical Research Ethics Committee of Ankara University Faculty of Medicine [Initial approval number: İ09-714-24 (01.11.2024), revision approval number: İ04-342-25 (05.05.2025)], in accordance with the Declaration of Helsinki and Good Clinical Practice (GCP) guidelines. The study is conducted and reported according to the ESMO Guidance for Reporting Oncology Real-World Evidence (ESMO-GROW)[1], following the Strengthening the Reporting of Observational Studies in Epidemiology (STROBE) statement[2] **(Supplementary documents: ESMO-GROW checklist, STROBE checklist).** All authors had access to the study data and reviewed and approved the final manuscript.

1.2. Eligibility of patients

Patients aged 18 years or older, with histologically confirmed and surgically resected BTC, including intrahepatic, perihilar, distal CCA, and gallbladder cancer, were eligible for inclusion, provided they had a follow-up duration of at least 2 months after initiation of adj-ChT (assuming a first possible assessment) and no more than 150 months (12.5 years, based on initial pilot data assessment showing extreme cases beyond 12.5 years follow-up). Patients diagnosed between 2010-2024 were recruited from hospitals with an oncology unit across all geographic regions of Türkiye, including the Aegean, Marmara, Central Anatolia, Mediterranean, Black Sea, Eastern Anatolia, and Southeastern Anatolia regions.

Exclusion criteria included histological diagnoses other than adenocarcinoma, ampullary carcinoma, R2 surgical resection, absence of adj-ChT, receipt of adjuvant RT or chemotherapy alone without an adj-ChT regimen, administration of neoadjuvant therapy before surgery, missing survival data, poor data quality based on data quality management strategies, or follow-up less than 2 months or exceeding 150 months.

1.3. Data collection

The following variables were collected for each patient: sex, age at diagnosis, Eastern Cooperative Oncology Group performance status (ECOG-PS), primary tumor location, T stage, lymph node status, resection margin status (R0 or R1), histological grade, presence of lymphovascular invasion (LVI) and/or perineural invasion (PNI), type of adj-ChT regimen, date of chemotherapy initiation, completion status of the planned treatment cycles, requirement for dose reduction, receipt of additional RT or chemoradiotherapy (CRT), and occurrence of grade 3–4 adverse events as defined by the Common Terminology Criteria for Adverse Events version 5.0 (CTCAE v5.0) during adj-ChT. Outcomes recorded included the date of disease recurrence (defined

based on imaging with or without biopsy) or death from any cause, and the date of the last follow-up visit. All clinical, radiological, and pathological data were obtained from institutional records based on local diagnostic and treatment protocols.

1.4. Data Quality Management

A prespecified data quality management strategy was implemented to ensure the accuracy, completeness, consistency, uniqueness, timeliness, and validity of the dataset. Centralized data review was conducted independently by two investigators (E.A. and M.K.). Each data entry was examined for coding accuracy, numerical format, and typographical errors. Completeness of required variables was verified for each patient, and cases missing survival data were excluded in accordance with the study's exclusion criteria. Other missing data points were recorded as "unknown/not available" and included in descriptive statistics and multivariable analyses.

To ensure consistency, data from different centers were standardized, while records within the same center were reviewed to prevent duplication, thereby ensuring uniqueness. Timeliness was verified against the study's inclusion criteria, particularly the minimum and maximum follow-up periods. Outliers, such as cases with unusually early or late recurrence, or prolonged survival despite recurrence, were flagged and clarified with the respective participating center. Any identified data entry errors were corrected following confirmation from the originating site. Cases with unresolved or unverifiable discrepancies were classified as "low-quality data" and excluded from the analysis.

1.5. Outcomes

Disease and treatment characteristics of the entire study population and the most common adj-ChT regimen groups were summarized. The primary endpoints of the

study were RFS and OS. RFS and OS outcomes were analysed across different adj-ChT regimens compared to Cape. To address prognostic factor differences and selection bias between adj-ChT regimens, multivariable Cox regression analyses were performed, adjusting for age, sex, era, performance score, primary tumor location, T stage, lymph node status, resection margin (R0 vs R1), tumor grade, presence of lymphovascular invasion (LVI), perineural invasion (PNI), and receipt of adjuvant RT or CRT. Additionally, propensity scoring by inverse probability of treatment weighting (IPTW) was performed for the most common four adj-ChT regimens (Gem-Cis vs Cape, Gem-Cape vs. Cape, Gem vs Cape) to further address selection bias.

Secondary endpoints included comparisons of RFS and OS among the most commonly used adj-ChT regimens within prespecified subgroups, including primary tumor site, resection status, and lymph node involvement. Subgroup analyses were adjusted for the same set of covariates as the primary analysis. An additional secondary objective was to analyse 2-year RFS and OS of the most common adj-ChT regimens, as assessed by restricted mean survival time (RMST).

RFS was defined as the time from the initiation of adj-ChT to the date of documented recurrence or death from any cause, whichever occurred first. OS was defined as the time from the initiation of adj-ChT to death from any cause.

1.6. Statistical analyses

Continuous variables were reported as median values with corresponding minimum and maximum ranges, while categorical variables were presented as percentages.

Median follow-up was calculated using the reverse Kaplan-Meier method. Univariable unadjusted comparisons of RFS and OS across chemotherapy regimens were performed using the log-rank test and visualized with Kaplan–Meier survival curves.

Missing data were handled using multiple imputation with chained equations (MICE). Twenty-five imputed datasets were generated using the fully conditional specification. The imputation model included treatment assignments, outcome variables, and all covariates used in the propensity score model and multivariable analyses. Analyses were performed within each imputed dataset and pooled using Rubin's rules. Multivariable Cox regression analyses and IPTW analyses were performed after multiple imputations.

Multivariable adjusted analyses of RFS and OS were conducted using Cox proportional hazards regression models, with results presented as hazard ratios and 95% confidence intervals in forest plots. Joint (global) p-values in multivariable analyses were obtained through the Wald test.

Inverse probability of treatment weighting (IPTW) based on propensity scores was used for the weighted Cox proportional hazards regression model to compare adj-ChTs with Cape[3]. The propensity scores were calculated through logistic regression using prognostic variables [age, sex, era, performance score, primary tumor location, T stage, lymph node status, resection margin (R0 vs R1), tumor grade, presence of lymphovascular invasion (LVI), perineural invasion (PNI), and receipt of adjuvant RT or CRT]. To improve model stability and reduce the influence of extreme weights, IPTW values greater than 10 were truncated at 10 (ranging from upper 0% to a maximum 2% of weights among the multiple imputation individual datasets). Propensity score estimation and IPTW-weighted Cox models were performed within each imputed dataset, and estimates were pooled using Rubin's rules. Covariate

balance between treatment groups before and after weighting was assessed using standardized mean differences (SMDs) and presented as Love plots, with an absolute SMD <0.1 considered indicative of adequate balance. In instances where residual imbalance remained (SMD between 0.1 and 0.2), the corresponding covariate was additionally included in the weighted outcome models as an adjustment variable, as double adjustment after IPTW is recommended when residual imbalance persists.

Two-year RFS and OS outcomes were further analyzed using the RMST method[4]. Prespecified subgroup analyses were performed to compare RFS and OS between chemotherapy regimens (compared to Cape) based on lymph node status, resection margin status (R0 vs R1), and primary tumor anatomical location. All subgroup comparisons were conducted using multivariable Cox regression analyses, adjusting for relevant covariates including age, sex, performance score, tumor location, T stage, lymph node status, resection status, grade, LVI, PNI, and RT/CRT use.

All statistical tests were two-sided, with a significance threshold of $p < 0.05$. The analyses and visualization were performed using R Version 4.4.2, RStudio, and MedCalc Statistical Software version 22.026 (MedCalc Software Ltd, Ostend, Belgium).

2. Figures

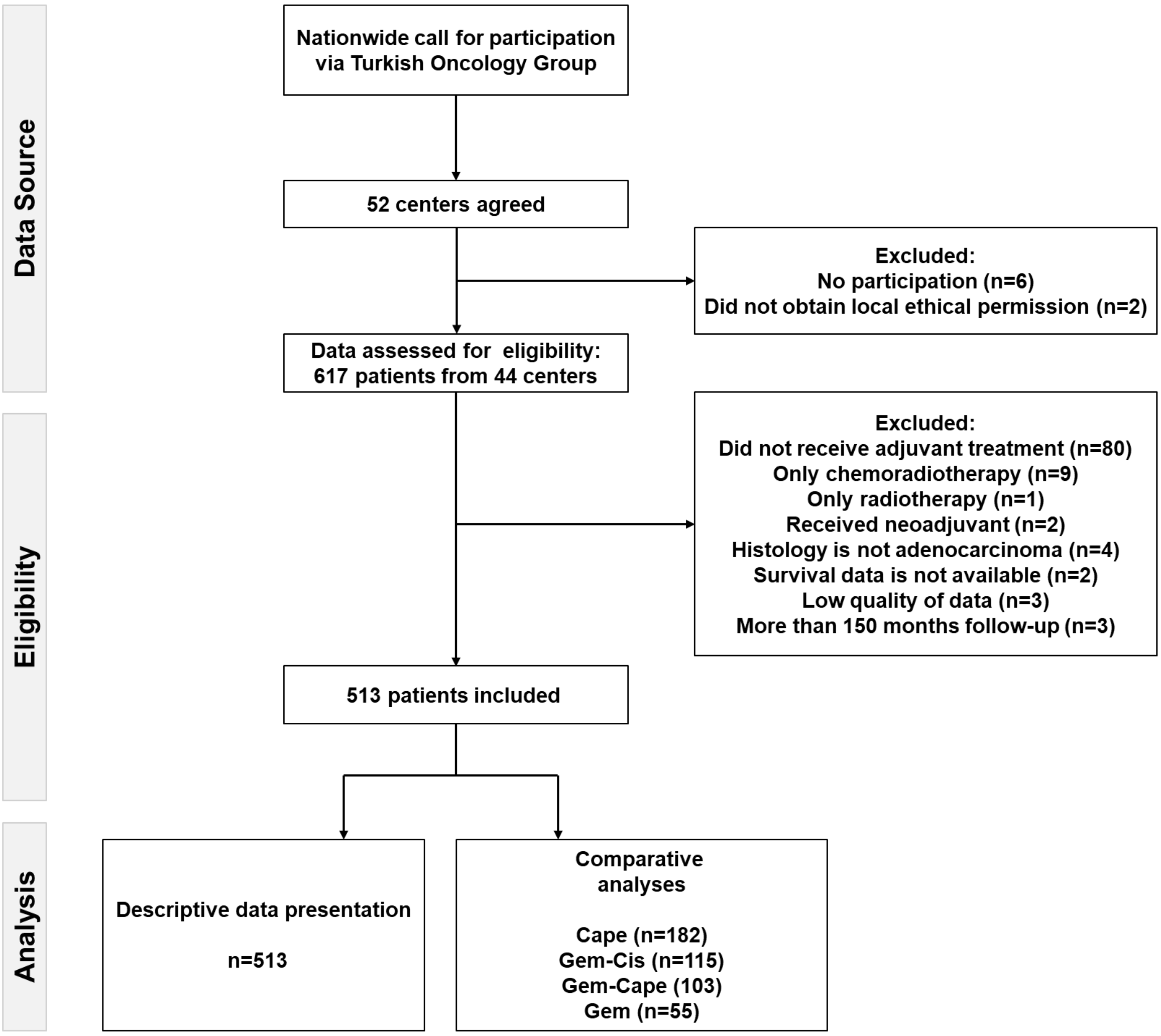

Figure S1. ESMO-GROW flowchart for real-world evidence studies in oncology: Formation and selection of the study population. The study protocol was approved at the Turkish Oncology Group annual meeting (2024). The oncology centers in Türkiye were called for participation via online notification. *Cape: Capecitabine, Gem-Cis: Gemcitabine-cisplatin, Gem-Cape: Gemcitabine-capecitabine, Gem: Gemcitabin*

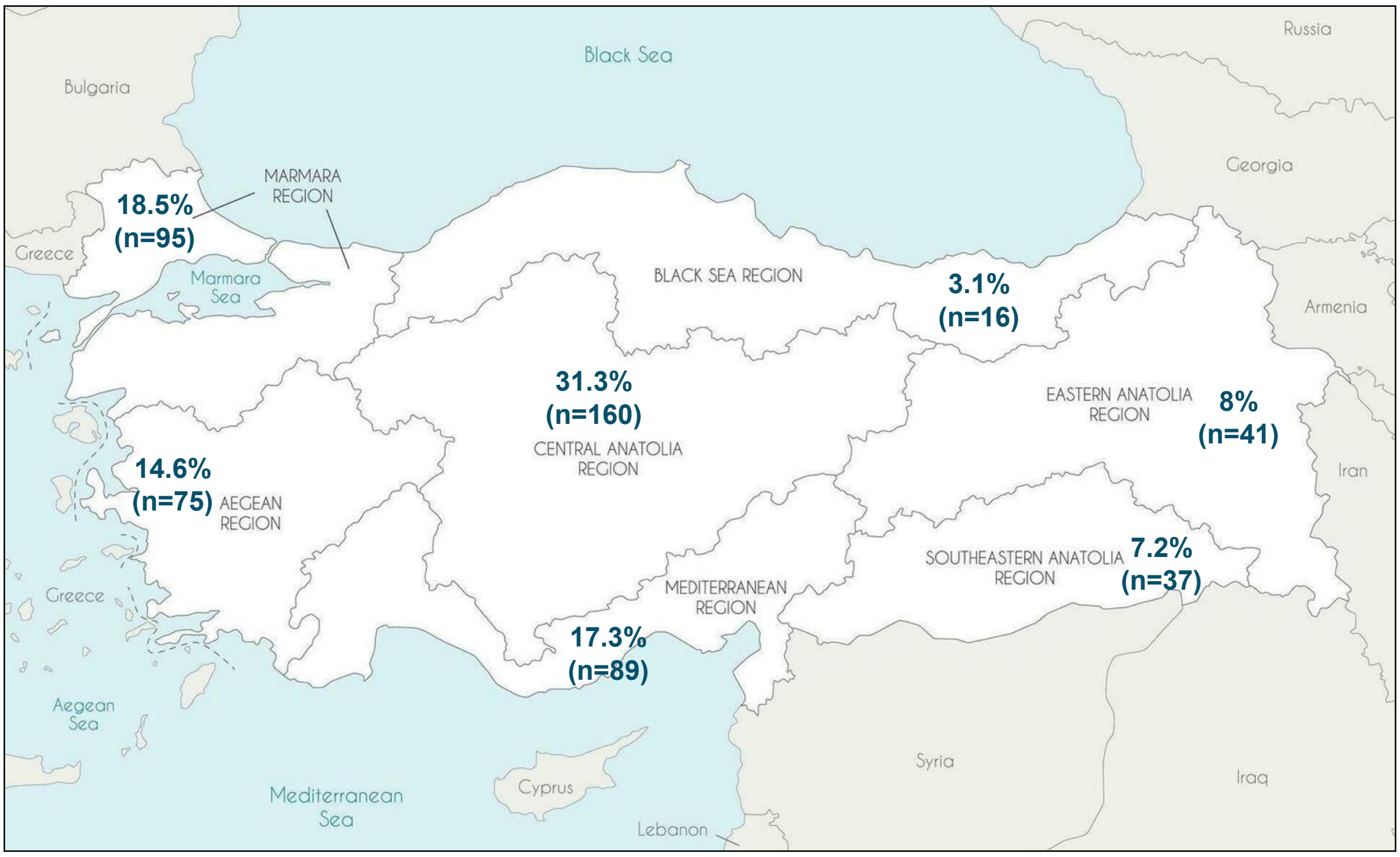

Figure S2. Distribution of patients (n=513) across Türkiye. Türkiye bridges the continents of Europe and Asia as shown in the figure. It comprises seven geographical regions named on the map. The study population was distributed throughout these regions from Europe to Asia.

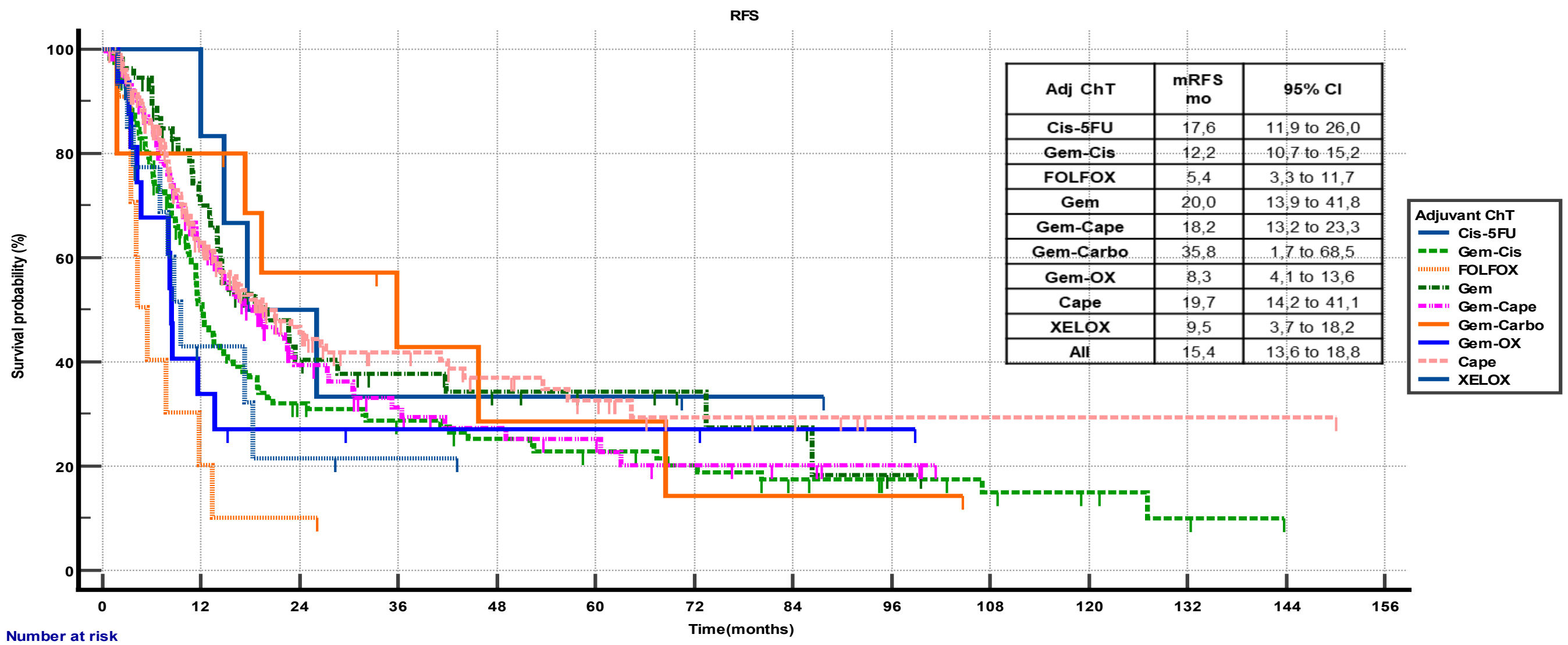

Adj ChT	mRFS mo	95% CI
Cis-5FU	17,6	11,9 to 26,0
Gem-Cis	12,2	10,7 to 15,2
FOLFOX	5,4	3,3 to 11,7
Gem	20,0	13,9 to 41,8
Gem-Cape	18,2	13,2 to 23,3
Gem-Carbo	35,8	1,7 to 68,5
Gem-OX	8,3	4,1 to 13,6
Cape	19,7	14,2 to 41,1
XELOX	9,5	3,7 to 18,2
All	15,4	13,6 to 18,8

Number at risk

Group	0	12	24	36	48	60	72	84	96	108	120	132	144	156
Group: Cis-5FU	6	5	3	2	2	2	1	1	0	0	0	0	0	0
Group: Gem-Cis	115	51	30	25	21	18	15	11	8	6	4	2	0	0
Group: FOLFOX	12	2	1	0	0	0	0	0	0	0	0	0	0	0
Group: Gem	55	33	16	12	9	7	5	4	1	0	0	0	0	0
Group: Gem-Cape	103	56	26	17	13	11	7	5	3	0	0	0	0	0
Group: Gem-Carbo	10	8	5	3	2	2	1	1	1	0	0	0	0	0
Group: Gem-OX	16	5	3	2	2	2	2	1	1	0	0	0	0	0
Group: Cape	182	84	41	28	19	14	7	6	1	1	1	1	1	0
Group: XELOX	14	4	2	1	0	0	0	0	0	0	0	0	0	0

Figure S3. Recurrence-free survivals (RFS) according to the adjuvant chemotherapy regimens: Kaplan-Meier plots and median RFS in months. *Adj Cht: Adjuvan chemotherapy, mRFS: median RFS, mo: months, Cis-5FU: cisplatin-5fluouracil, Gem-Cis: Gemcitabin-cisplatin, FOLFOX: folinic acid, fluorouracil, and oxaliplatin, Gem: Gemcitabine, Gem-Cape: Gemcitabine-capecitabine, Gem-Carbo:Gemcitabin-carboplatin, Gem-OX: Gemcitabin-Oxaliplatin, Cape: Capecitabine, XELOX: oxaliplatin-capecitabine*

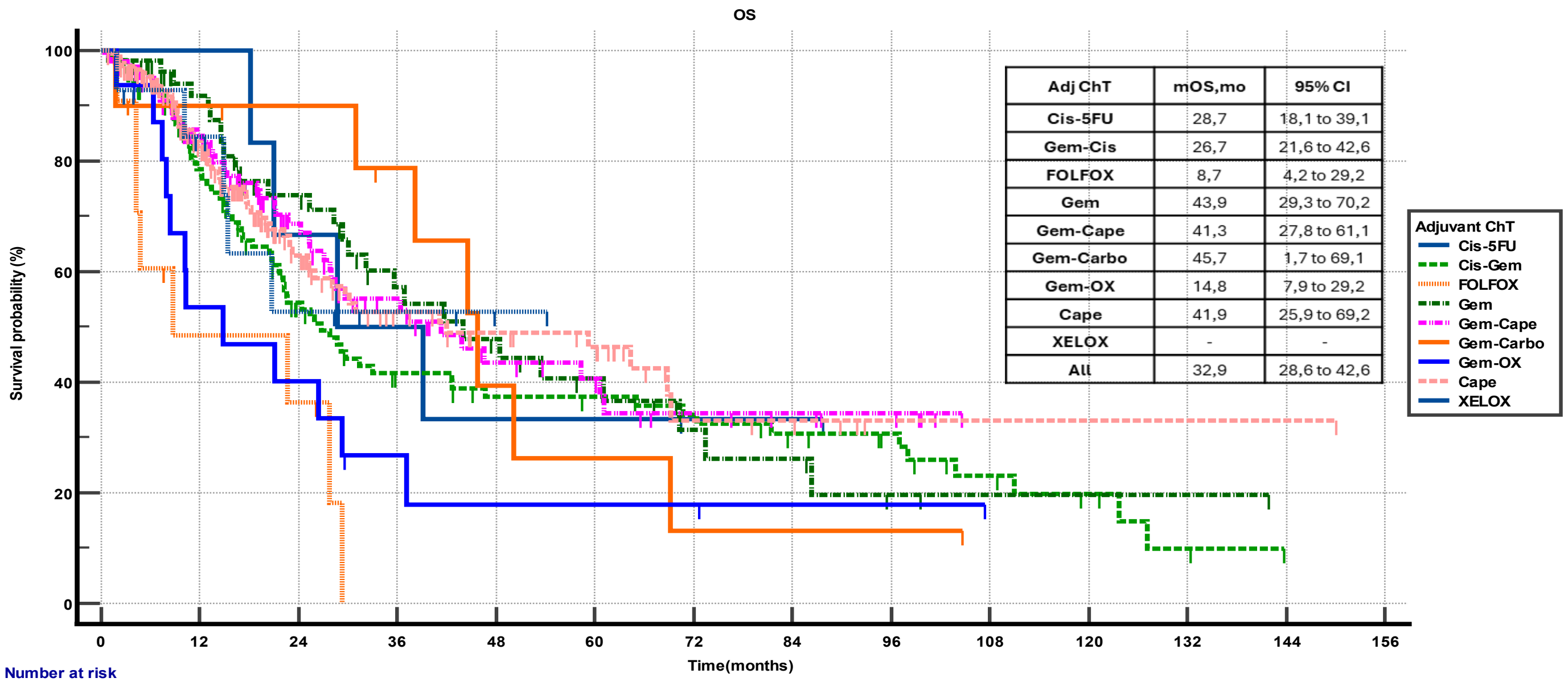

Adj ChT	mOS,mo	95% CI
Cis-5FU	28,7	18,1 to 39,1
Gem-Cis	26,7	21,6 to 42,6
FOLFOX	8,7	4,2 to 29,2
Gem	43,9	29,3 to 70,2
Gem-Cape	41,3	27,8 to 61,1
Gem-Carbo	45,7	1,7 to 69,1
Gem-OX	14,8	7,9 to 29,2
Cape	41,9	25,9 to 69,2
XELOX	-	-
All	32,9	28,6 to 42,6

Number at risk	0	12	24	36	48	60	72	84	96	108	120	132	144	156
Group: Cis-5FU	6	6	4	3	2	2	1	1	0	0	0	0	0	0
Group: Cis-Gem	115	74	44	30	25	24	21	16	13	8	5	2	0	0
Group: FOLFOX	12	4	3	0	0	0	0	0	0	0	0	0	0	0
Group: Gem	55	42	29	19	14	10	6	5	2	1	1	1	0	0
Group: Gem-Cape	103	74	42	27	17	14	9	7	5	0	0	0	0	0
Group: Gem-Carbo	10	9	8	6	3	2	1	1	1	0	0	0	0	0
Group: Gem-OX	16	8	6	3	2	2	2	1	1	0	0	0	0	0
Group: Cape	182	110	52	30	22	18	7	6	1	1	1	1	1	0
Group: XELOX	14	9	5	3	1	0	0	0	0	0	0	0	0	0

Figure S4. Overall survival (OS) according to the adjuvant chemotherapy regimens: Kaplan-Meier plots and median OS in months. *Adj Cht: Adjuvan chemotherapy, mRFS: median RFS, mo: months, Cis-5FU: cisplatin-5fluouracil, Gem-Cis: Gemcitabin-cisplatin, FOLFOX: folinic acid, fluorouracil, and oxaliplatin, Gem: Gemcitabine, Gem-Cape: Gemcitabine-capecitabine, Gem-Carbo:Gemcitabin-carboplatin, Gem-OX: Gemcitabin-Oxaliplatin, Cape: Capecitabine, XELOX: oxaliplatin-capecitabine*

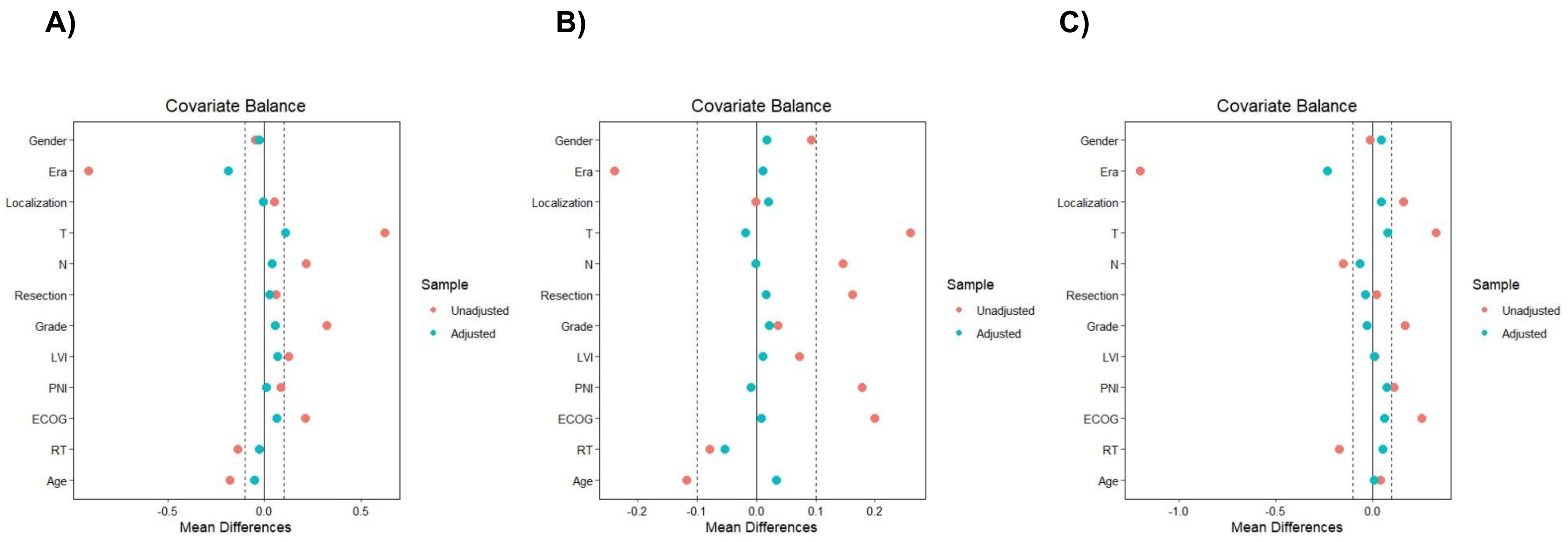

Figure S5. Love plot presentations of covariate balance evaluation by standardized mean difference (threshold <0.1) after inverse probability of treatment weighting (IPTW). A) Gem-Cis vs. Cape, B) Gem-Cape vs Cape, C) Gem vs Cape

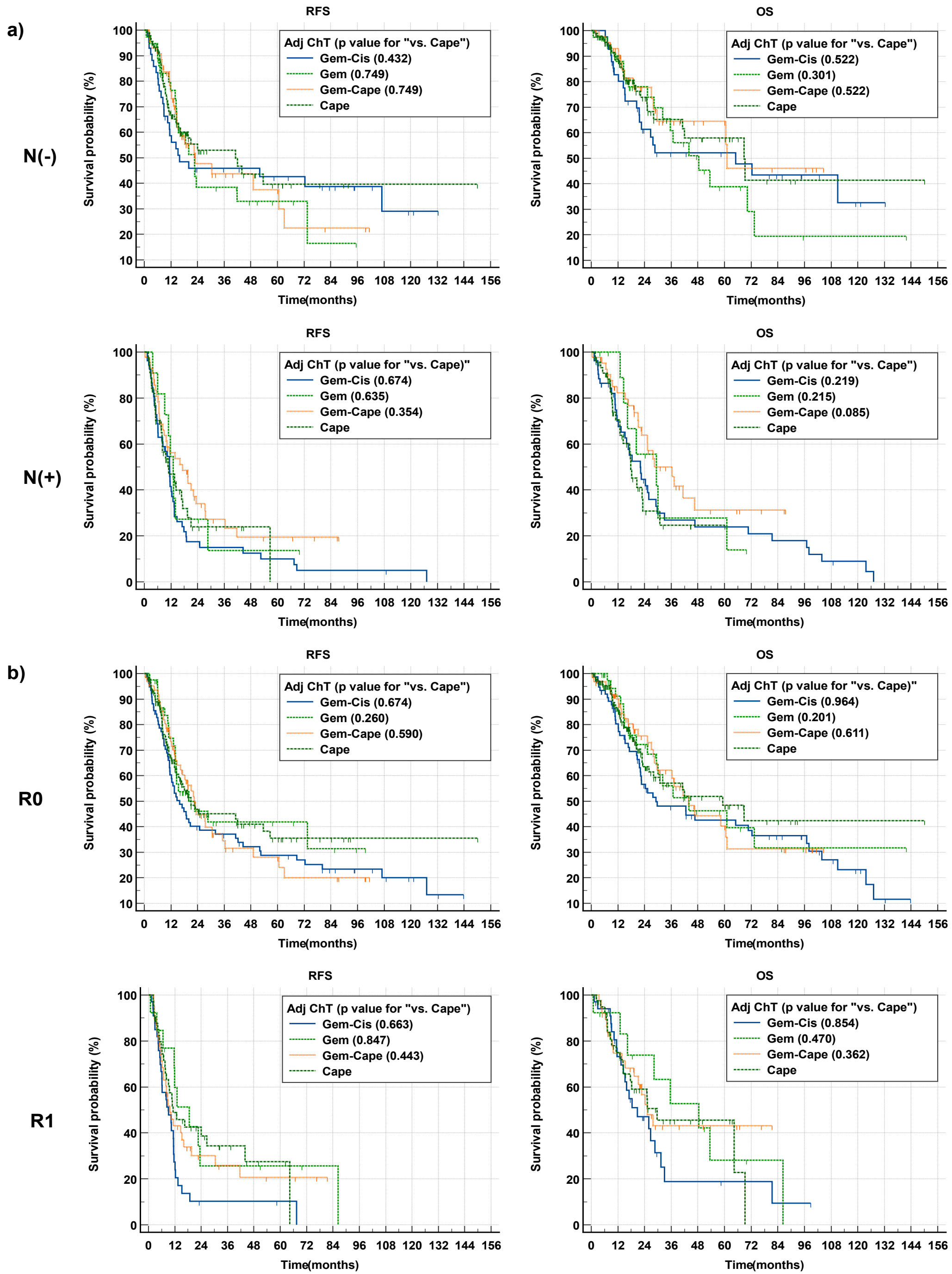
a)
N(-)
N(+)
b)
R0
R1
RFS
OS
Survival probability (%)
Time(months)
0
12
24
36
48
60
72
84
96
108
120
132
144
156
Adj ChT (p value for "vs. Cape")
Gem-Cis (0.432)
Gem (0.749)
Gem-Cape (0.749)
Cape
Adj ChT (p value for "vs. Cape")
Gem-Cis (0.522)
Gem (0.301)
Gem-Cape (0.522)
Cape
Adj ChT (p value for "vs. Cape)"
Gem-Cis (0.674)
Gem (0.635)
Gem-Cape (0.354)
Cape
Adj ChT (p value for "vs. Cape")
Gem-Cis (0.219)
Gem (0.215)
Gem-Cape (0.085)
Cape
Adj ChT (p value for "vs. Cape")
Gem-Cis (0.674)
Gem (0.260)
Gem-Cape (0.590)
Cape
Adj ChT (p value for "vs. Cape)"
Gem-Cis (0.964)
Gem (0.201)
Gem-Cape (0.611)
Cape
Adj ChT (p value for "vs. Cape")
Gem-Cis (0.663)
Gem (0.847)
Gem-Cape (0.443)
Cape
Adj ChT (p value for "vs. Cape")
Gem-Cis (0.854)
Gem (0.470)
Gem-Cape (0.362)
Cape

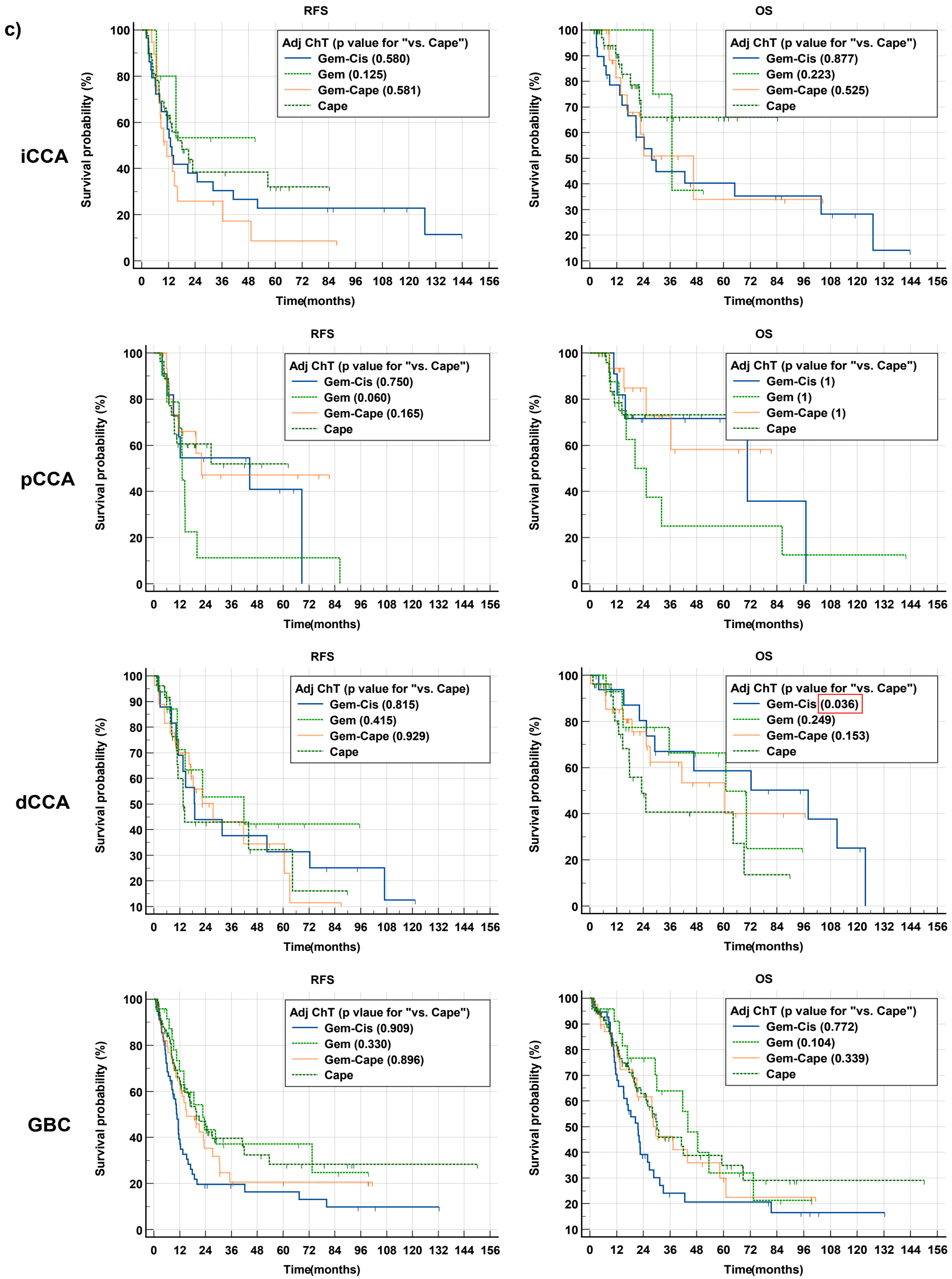

c)
iCCA
pCCA
dCCA
GBC
RFS
OS
Survival probability (%)
Time(months)
Adj ChT (p value for "vs. Cape")
Gem-Cis (0.580)
Gem (0.125)
Gem-Cape (0.581)
Cape
Adj ChT (p value for "vs. Cape")
Gem-Cis (0.877)
Gem (0.223)
Gem-Cape (0.525)
Cape
Adj ChT (p value for "vs. Cape")
Gem-Cis (0.750)
Gem (0.060)
Gem-Cape (0.165)
Cape
Adj ChT (p value for "vs. Cape")
Gem-Cis (1)
Gem (1)
Gem-Cape (1)
Cape
Adj ChT (p value for "vs. Cape")
Gem-Cis (0.815)
Gem (0.415)
Gem-Cape (0.929)
Cape
Adj ChT (p value for "vs. Cape")
Gem-Cis (0.036)
Gem (0.249)
Gem-Cape (0.153)
Cape
Adj ChT (p vlaue for "vs. Cape")
Gem-Cis (0.909)
Gem (0.330)
Gem-Cape (0.896)
Cape
Adj ChT (p value for "vs. Cape")
Gem-Cis (0.772)
Gem (0.104)
Gem-Cape (0.339)
Cape

Figure S6. Prespecified adjusted subgroup analyses a) RFS and OS for lymph node (N) negative and positive groups, b) RFS and OS for R0 and R1 resection groups, c) RFS and OS for anatomical subtypes. All p-values are compared to Cape and adjusted by age, gender, ECOG, T, N, R, grade, localization, and (chemo)radiotherapy. *iCCA: intrahepatic cholangiocarcinoma, pCCA: perihilar cholangiocarcinoma, dCCA: distal cholangiocarcinoma, GBC: Gallbladder cancer, Adj ChT: Adjuvant chemotherapy, Cape: Capecitabine, Gem-Cis: Gemcitabine-cisplatin, Gem: Gemcitabine, Gem-Cape: Gemcitabine*

3. Tables

Table S1. Completion rates between chemotherapy regimens

**Cape**	**Gem-Cis**	**Gem-Cape**	**Gem**	**Gem-OX**	**XELOX**	**FOLFOX**	**Gem-Carbo**	**Cis-5FU**	***P***
78.8%	76.9%	79.8%	78.2%	93.7%	66.7%	75%	80%	83.3%	0.766

Table S2. Comparison of characteristics between patients who received and did not receive radiotherapy (RT or CRT)

Characteristic	Comparison (Received vs Not received)	P
**Age**	>65: 34.7% vs. 43.1%	**0.036**
**ECOG-PS**	0: 33.2% vs 29.4%<br>1: 55.9% vs 58.4%	0.569
**Localization**	Intrahepatic: 18.1% vs 22.5%<br>Perihilar: 16.6% vs 11.9%<br>Distal: 18.7% vs 17.2%<br>Gallbladder: 40.4% vs 43.7%<br>Undetermined: 6.2% vs 4.7%	0.399
**T stage**	T3: 37.8% vs. 32.2%<br>T4: 6.7% vs. 6.6%	0.285
**N positivity**	43.5% vs 26.6%	**<0.001**
**R1 resection**	35.2% vs 23.4%	**0.004**
**Grade**	2: 49.7% vs 47.2%<br>3: 14.0% vs 14.7%	0.950
**LVI**	54.2% vs 47.5%	0.249
**PNI**	60.1% vs 44.3%	**0.002**

Table S3. Comparison of characteristics between patients who received CRT and RT

**Characteristic**	**Comparison (CRT vs RT)**	**P**
**Age**	>65: 27.6% vs. 37.8%	0.173
**ECOG-PS**	0: 34.5% vs 32.6%<br>1: 56.9% vs 55.6%	0.529
**Localization**	Intrahepatic: 12.1% vs 20.7%<br>Perihilar: 15.5% vs 17.0%<br>Distal: 19% vs 18.5%<br>Gallbladder: 36.2% vs 42.2%<br>Undetermined: 17.2% vs 1.5%	**0.001**
**T stage**	T3: 34.5% vs. 39.3%<br>T4: 5.2% vs. 7.4%	0.732
**N positivity**	32.8% vs 48.1%	**0.041**
**R1 resection**	39.7% vs 33.3%	0.581
**Grade**	2: 46.6% vs 51.1%<br>3: 13.8% vs 14.1%	0.114
**LVI**	51.7% vs 55.2%	0.740
**PNI**	63.8% vs 58.5%	0.787

Table S4. 2-year restricted mean survival times (RMST) for recurrence-free survival (RFS) and overall survival (OS)

Months*, mean (95% CI)	Cape	Gem-Cis	Gem-Cape	Gem
**2-year RFS**	16.4 (15.1-17.7)	13.9 (12.3-15.4)	16.0 (14.4-17.6)	17.1 (15.0-19.1)
**2-year OS**	19.9 (18.8-20.9)	18.9 (17.6- 20.3)	20.2 (18.9-21.5)	21.1 (19.6-22.6)

Gem-Cis: Gemcitabin-cisplatin, Gem: Gemcitabine, Gem-Cape: Gemcitabine-capecitabine, Cape: Capecitabine, RFS: recurrence-free survival, OS: overall survival ***The results should be interpreted considering baseline characteristic differences between treatment groups.**

Table S5. 2-year restricted mean survival time comparison for RFS and OS (Capecitabine vs other regimens)*

CAPE VS.	2-year RFS		2-year OS	
	Difference (months) 95% CI	*P*	Difference (months) 95% CI	*P*
**Gem-Cis**	**2.55 (0.54 to 4.57)**	**0.012**	0.94 (-0.73 to 2.61)	0.270
**Gem-Cape**	0.43 (-1.64 to 2.51)	0.680	-0.32 (-1.98 to 1.33)	0.701
**Gem**	-0.61 (-3.03 to 1.79)	0.615	-1.17 (-2.99 to 0.64)	0.205
**Gem-OX**	**4,91 (0.59 to 9.23)**	**0.025**	**4.39 (0.29 to 8.48)**	**0.035**
**Xelox**	4,01 (-0,61 to 8.63)	0.089	0.90 (-2.93 to 4.75)	0.642
**Folfox**	**8.50 (4.30 to 12.70)**	**<0.001**	5.90 (-0.12 to 11.92)	0.054
**Gem-Carbo**	-1.76 (-7.27 to 3.73)	0.529	-1.84 (-6.05 to 2.37)	0.391
**Cis-5FU**	-2.89 (-7.02 to 1.23)	0.168	**-2.58 (-4.57 to -0.59)**	**0.010**

Cis-5FU: cisplatin-5fluouracil, Gem-Cis: Gemcitabin-cisplatin, FOLFOX: folinic acid, fluorouracil, and oxaliplatin, Gem: Gemcitabine, Gem-Cape: Gemcitabine-capecitabine, Gem-Carbo:Gemcitabin-carboplatin, Gem-OX: Gemcitabin-Oxaliplatin, Cape: Capecitabine, XELOX: oxaliplatin-capecitabine, RFS: recurrence-free survival, OS: overall survival, CI: confidence interval, ***The results present univariable unadjusted comparisons; therefore, they should be interpreted cautiously, considering baseline prognostic differences in treatment groups.**

4. References

[1] Castelo-Branco L, Pellat A, Martins-Branco D, Valachis A, Derksen JWG, Suijkerbuijk KPM, et al. ESMO Guidance for Reporting Oncology Real-World Evidence (GROW). Annals of Oncology 2023;34:1097-1112.

[2] von Elm E, Altman DG, Egger M, Pocock SJ, Gøtzsche PC, Vandenbroucke JP. The Strengthening the Reporting of Observational Studies in Epidemiology (STROBE) statement: guidelines for reporting observational studies. Journal of Clinical Epidemiology 2008;61:344-349.

[3] Cole SR, Hernán MA. Constructing inverse probability weights for marginal structural models. Am J Epidemiol 2008;168:656-664.

[4] Royston P, Parmar MK. Restricted mean survival time: an alternative to the hazard ratio for the design and analysis of randomized trials with a time-to-event outcome. BMC Med Res Methodol 2013;13:152.

Appendix 1. STROBE Statement—checklist of items that should be included in reports of observational studies

	Item No /*Page*	Recommendation
**Title and abstract**	1/*1-2*	(*a*) Indicate the study's design with a commonly used term in the title or the abstract
		(*b*) Provide in the abstract an informative and balanced summary of what was done and what was found
**Introduction**		
Background/rationale	2/*3*	Explain the scientific background and rationale for the investigation being reported
Objectives	3/*4*	State specific objectives, including any prespecified hypotheses
**Methods**		
Study design	4/*4*	Present key elements of study design early in the paper
Setting	5/*5-6-7*	Describe the setting, locations, and relevant dates, including periods of recruitment, exposure, follow-up, and data collection
Participants	6/*5*	(*a*) *Cohort study*—Give the eligibility criteria, and the sources and methods of selection of participants. Describe methods of follow-up<br>*Case-control study*—Give the eligibility criteria, and the sources and methods of case ascertainment and control selection. Give the rationale for the choice of cases and controls<br>*Cross-sectional study*—Give the eligibility criteria, and the sources and methods of selection of participants
		(*b*) *Cohort study*—For matched studies, give matching criteria and number of exposed and unexposed<br>*Case-control study*—For matched studies, give matching criteria and the number of controls per case
Variables	7/*6-7-8*	Clearly define all outcomes, exposures, predictors, potential confounders, and effect modifiers. Give diagnostic criteria, if applicable
Data sources/ measurement	8*/*7-8*	For each variable of interest, give sources of data and details of methods of assessment (measurement). Describe comparability of assessment methods if there is more than one group
Bias	9/*6*	Describe any efforts to address potential sources of bias
Study size	10/*4*	Explain how the study size was arrived at
Quantitative variables	11/*7-8*	Explain how quantitative variables were handled in the analyses. If applicable, describe which groupings were chosen and why
Statistical methods	12/*8*	(*a*) Describe all statistical methods, including those used to control for confounding
		(*b*) Describe any methods used to examine subgroups and interactions
		(*c*) Explain how missing data were addressed
		(*d*) *Cohort study*—If applicable, explain how loss to follow-up was addressed<br>*Case-control study*—If applicable, explain how matching of cases and controls was addressed<br>*Cross-sectional study*—If applicable, describe analytical methods taking account of sampling strategy
		(*e*) Describe any sensitivity analyses

Continued on next page

**Results**		
Participants	13*/*9-10-20*	(a) Report numbers of individuals at each stage of study—eg numbers potentially eligible, examined for eligibility, confirmed eligible, included in the study, completing follow-up, and analysed
		(b) Give reasons for non-participation at each stage
		(c) Consider use of a flow diagram
Descriptive data	14*/*9-10*	(a) Give characteristics of study participants (eg demographic, clinical, social) and information on exposures and potential confounders
		(b) Indicate number of participants with missing data for each variable of interest
		(c) *Cohort study*—Summarise follow-up time (eg, average and total amount)
Outcome data	15*/*10-11*	*Cohort study*—Report numbers of outcome events or summary measures over time
		Case-control study—Report numbers in each exposure category, or summary measures of exposure
		Cross-sectional study—Report numbers of outcome events or summary measures
Main results	16/*10-11-12*	(*a*) Give unadjusted estimates and, if applicable, confounder-adjusted estimates and their precision (eg, 95% confidence interval). Make clear which confounders were adjusted for and why they were included
		(*b*) Report category boundaries when continuous variables were categorized
		(*c*) If relevant, consider translating estimates of relative risk into absolute risk for a meaningful time period
Other analyses	17/*12*	Report other analyses done—eg analyses of subgroups and interactions, and sensitivity analyses
**Discussion**		
Key results	18/*13*	Summarise key results with reference to study objectives
Limitations	19/*18*	Discuss limitations of the study, taking into account sources of potential bias or imprecision. Discuss both direction and magnitude of any potential bias
Interpretation	20/*13 to 18*	Give a cautious overall interpretation of results considering objectives, limitations, multiplicity of analyses, results from similar studies, and other relevant evidence
Generalisability	21 /*18*	Discuss the generalisability (external validity) of the study results
**Other information**		
Funding	22/*19*	Give the source of funding and the role of the funders for the present study and, if applicable, for the original study on which the present article is based

*Give information separately for cases and controls in case-control studies and, if applicable, for exposed and unexposed groups in cohort and cross-sectional studies.

Note: An Explanation and Elaboration article discusses each checklist item and gives methodological background and published examples of transparent reporting. The STROBE checklist is best used in conjunction with this article (freely available on the Web sites of PLoS Medicine at http://www.plosmedicine.org/, Annals of Internal Medicine at http://www.annals.org/, and Epidemiology at http://www.epidem.com/). Information on the STROBE Initiative is available at www.strobe-statement.org.

Appendix 2

ESMO GROW

GUIDANCE FOR REPORTING ONCOLOGY REAL-WORLD EVIDENCE

ESMO-GROW Checklist for Authors and Reviewers

This checklist integrates all ESMO-GROW recommendation and could be used by authors and reviewers when assessing the reporting standards of a real-world evidence study in Oncology.

For the the ESMO-GROW checklist, the following criteria are considered:
"Yes, fully reported" – The recommendation is adequately considered.
"Yes, partially reported" – The recommendation is considered, but some important details are missing.
"Not reported" – The recommendation is applicable for the case, but it was not considered.
"Not applicable" – The recommendation is not applicable for this study.

Name of Author/Reviewer: **Date:**

Title of Manuscript or Identifier:

Recommendations	Yes, fully reported	Yes, partially reported	Not reported	Not applicable
**1. Title**				
1.1: Concisely include relevant key terms referring to the study type, study population, objectives, data sources and outcomes, depending on the study. Consider including the terms 'real-world' or 'observational'	●	○	○	○
**2. Introduction**				
2.1: Explain the scientific rationale for the research question(s), providing concise background information on previous core evidence from systematic reviews, meta-analyses, clinical trials and/or real-world evidence studies	●	○	○	○
2.2: Identify the gaps in evidence and explain why and how they can be suitably addressed by real-world evidence research. Specify the new evidence that is expected from the current study	●	○	○	○
2.3: Briefly introduce the aim(s) of the study	●	○	○	○
**3. Methods**				
Study objective(s), design, data sources and variables				
3.1: Provide the study research question(s) including a description of the patients or the object under study and the target outcome(s)	●	○	○	○
3.2: Provide the study objective(s) and consider classifying the type of research as descriptive and/or analytical (explanatory or predictive)	●	○	○	○
3.3: Provide relevant information to describe and classify the study design used to address the research question	●	○	○	○
3.4: Give a clear definition of the eligibility criteria used to select the patients or objects under study, particularly regarding cancer-related aspects	●	○	○	○
3.5: Report the specific type and purpose of real-world data source(s) used, providing a detailed description and the reason(s) why the source was considered appropriate for the study objectives	●	○	○	○
3.6: When multiple real-world data sources are used, provide details on interoperability, including identification of duplicated cases or data linkage from separate databases	○	●	○	○
3.7: Provide details and timings of source and study data management. Consider specifying methods of raw data collection, updates and completeness, data extraction, cleaning and/or quality controls and validation	●	○	○	○
3.8: Provide core details on database and/or study registration, governance, ownership, metadata and full data accessibility in the main text or supplementary material	●	○	○	○
3.9: Identify the data source of each core variable, its definition, if the variable was derived or coded, and describe how the derivation or coding was conducted and validated.	●	○	○	○
3.10: Specify the time points of core variables in relation to the cancer disease trajectory	●	○	○	○
3.11: Provide a complete list of core variables included in the study. Variables can be grouped as baseline characteristics, exposure and outcomes or endpoints	●	○	○	○
3.12: For biomarker-related studies, provide details on biomarker description, timing, and methods of assessment and analytical validation	○	○	○	●
Statistical analysis and artificial intelligence methods				
3.13: Summarise the main aspects of the statistical analysis	●	○	○	○
3.14: When applicable, provide details on the pre-planned sample size requirements and power of the study	○	○	○	●
3.15: Specify the pre-planned strategies to identify and mitigate the main sources of bias	●	○	○	○
3.16: Clearly distinguish prespecified from *post hoc* analyses, especially for subgroup analyses	●	○	○	○
3.17: Provide information on internal and external validity, as well as any sensitivity analyses	○	○	○	●
3.18: For analytical studies, the full version of the statistical analysis plan should be provided in the supplementary material, including a brief explanation of any amendments	○	●	○	○
3.19: When applicable, specify which machine learning, deep learning or alternative artificial intelligence method has been used	○	○	○	●
3.20: When reporting real-world data analysis with artificial intelligence (e.g. machine learning and deep learning) algorithms, include comprehensive aspects on data pre-processing techniques, feature engineering strategies and model development	○	○	○	●
3.21: Address the artificial intelligence model explainability and interpretability, and present the plan for integration into clinical practice, if applicable	○	○	○	●
3.22: When applicable, briefly describe the multidisciplinary team required for the study and explain how these needs were met	●	○	○	○

ESMO GROW

GUIDANCE FOR REPORTING ONCOLOGY REAL-WORLD EVIDENCE

ESMO-GROW Checklist for Authors and Reviewers

Recommendations	Yes, fully reported	Yes, partially reported	Not reported	Not applicable
**4. Results**				
4.1: Provide number of cases excluded or nonparticipating and reasons at each stage of sample selection, as well as numbers lost to follow-up. Compare the cases excluded with those included in the analyses. Illustrate this with a flowchart	◉	○	○	○
4.2: Describe the baseline characteristics of the cases included (e.g. clinico-demographic and tumour characteristics). The baseline characteristics of different groups under analysis should be compared, if applicable	◉	○	○	○
4.3: Report the results of the primary analysis of study outcomes. Briefly describe the results of exploratory analyses if relevant (prespecified and/or *post hoc*). Provide details of how readers can access the full results	◉	○	○	○
**5. Discussion and conclusions**				
Discussion				
5.1: Summarise the core results that address the primary research question(s) and objectively discuss the data in relation to the best available evidence on the topic. Avoid a convenient selection of literature to support a point	◉	○	○	○
5.2: Discuss the strengths and limitations of the current study, including the main biases, how the strategies applied contributed to bias avoidance or mitigation, and, if applicable, in which direction the authors estimate that residual bias may influence the core results of the study	◉	○	○	○
5.3: Discuss the generalisability of the study results and their potential implications for clinical practice, health policies or public health and for the generation of hypotheses for future research	◉	○	○	○
Conclusions				
5.4: Provide a balanced summary of core results relating to the primary research question and the main implications for clinical practice, health policies and/or public health. Suggest further research considering the remaining unmet needs and limitations from the reported study	◉	○	○	○
**6. Final considerations**				
6.1: Specify all relevant study sponsorship(s) as well as direct and/or indirect or in-kind funding	◉	○	○	○
6.2: Specify all relevant acknowledgements, author disclosures, individual contributions and other final considerations as per journal regulations	◉	○	○	○

Notes:

To access full manuscripts and for citations, please consider the following references and links:

- Castelo-Branco L et al. "ESMO Guidance for Reporting Oncology real-World evidence (GROW)". Ann Oncol 2023; 34: 10.1016/j.annonc.2023.10.001
- Castelo-Branco L et al. "ESMO Guidance for Reporting Oncology real-World evidence (GROW)". ESMO Real World Data & Digital Oncol 2023; 1: 10.1016/j.esmorw.2023.10.001

ESMO GOOD SCIENCE BETTER MEDICINE BEST PRACTICE
